# Brown Fat Determination and Development from Muscle Precursor Cells by Novel Action of Bone Morphogenetic Protein 6

**DOI:** 10.1371/journal.pone.0092608

**Published:** 2014-03-21

**Authors:** Ankur Sharma, Christine Huard, Cecile Vernochet, Daniel Ziemek, Kelly M. Knowlton, Edyta Tyminski, Theresa Paradis, Ying Zhang, Jessica E. C. Jones, David von Schack, Christopher T. Brown, Patrice M. Milos, Anthony J. Coyle, Frederic Tremblay, Robert V. Martinez

**Affiliations:** 1 Centers for Therapeutic Innovation, Pfizer Inc., Boston, Massachusetts, United States of America; 2 CVMED, Pfizer Inc., Cambridge, Massachusetts, United States of America; 3 Computational Sciences Center of Emphasis, Pfizer Inc, Cambridge, Massachusetts, United States of America; 4 BioTherapeutics Clinical Research, Pfizer Inc., Cambridge, Massachusetts, United States of America; 5 Global BioTherapeutics Technologies, Pfizer Inc., Cambridge, Massachusetts, United States of America; Graduate School of Medicine, the University of Tokyo, Japan

## Abstract

Brown adipose tissue (BAT) plays a pivotal role in promoting energy expenditure by the virtue of uncoupling protein-1 (UCP-1) that differentiates BAT from its energy storing white adipose tissue (WAT) counterpart. The clinical implication of “*classical*” BAT (originates from Myf5 positive myoblastic lineage) or the “*beige*” fat (originates through trans-differentiation of WAT) activation in improving metabolic parameters is now becoming apparent. However, the inducers and endogenous molecular determinants that govern the lineage commitment and differentiation of *classical* BAT remain obscure. We report here that in the absence of any forced gene expression, stimulation with bone morphogenetic protein 6 (BMP6) induces brown fat differentiation from skeletal muscle precursor cells of murine and human origins. Through a comprehensive transcriptional profiling approach, we have discovered that two days of BMP6 stimulation in C2C12 myoblast cells is sufficient to induce genes characteristic of brown preadipocytes. This developmental switch is modulated in part by newly identified regulators, Optineurin (Optn) and Cyclooxygenase-2 (Cox2). Furthermore, pathway analyses using the Causal Reasoning Engine (CRE) identified additional potential causal drivers of this BMP6 induced commitment switch. Subsequent analyses to decipher key pathway that facilitates terminal differentiation of these BMP6 primed cells identified a key role for Insulin Like Growth Factor-1 Receptor (IGF-1R). Collectively these data highlight a therapeutically innovative role for BMP6 by providing a means to enhance the amount of myogenic lineage derived brown fat.

## Introduction

Obesity remains a major clinical challenge that warrants discovery of effective therapeutic modalities to control this global epidemic. The revelation of brown adipose tissue (BAT) in adult humans that was once thought to be limited to rodents and human infants has ignited a new scientific vigor in the metabolic field. This affords the opportunity to explore and exploit the thermogenic potential of this mitochondria laden tissue for an enhanced and sustained body weight management with clinical implications in improving a patient's metabolic profile [1]–[6].

Numerous groups have recently shown that activating brown fat leads to improved insulin sensitivity, increased glucose disposal, enhanced triglyceride clearance and energy expenditure as heat that is distributed throughout the body in a perfusion dependent manner [7]–[9]. As brown adipocytes can use both cellular triglycerides and glucose as fuel for heat generation during the uncoupled respiration, finding ways to enhance brown fat amount and activity may serve as a plausible therapeutic option to manage obesity. Uncoupling protein-1 (UCP-1) serves as an important clinical marker for heat dissipating brown adipose tissue which differentiates it from the white adipose tissue that lacks UCP-1 expression and is primarily specialized in energy storage [4]. Developmentally, brown and white adipose tissues are derived from distinct cellular lineages. While white adipose tissue originates primarily from mesenchymal lineage, brown preadipocytes predominantly have a myogenic transcriptional gene signature [10], [11]. Moreover, lineage tracing experiments in murine models have revealed that “*classical” (*also termed as *“de novo”* or *“constitutive”)* brown fat cells are derived from a Myf5 positive cellular lineage that also gives rise to skeletal muscle tissue, a switch governed by transcription factor Prdm16 dependent mechanisms [12]–[14].

With the clinical ramifications of brown fat now becoming apparent, much attention has been given to mechanisms driving trans-differentiation of white adipocytes to a brown-like “*beige”* or *“brite*” phenotype [15]–[20]. However, the mechanisms underpinning *classical* brown fat differentiation from a Myf5 positive myogenic lineage remain poorly understood. Very few studies have provided insight into the precise molecular network governing muscle precursor cells to brown adipogenic lineage transition. Earlier studies identified key transcription factors: PPARγ, C/EBP α, and FMIP that can drive pan-adipogenic differentiation of muscle precursor cells [21], [22]; however the specificity of these molecular targets towards a brown fat lineage was not investigated. More recently, using ectopic expression studies in Myf5 positive murine skeletal muscle derived C2C12 cells, three groups have identified the specific roles of Prdm16, miR-193b-365 and Ebf2 in brown adipocyte lineage differentiation [13], [23], [24]. These studies collectively identify C2C12 cells as a viable tool conducive for investigations directed at understanding the mechanisms driving *classical* brown adipogenic differentiation cascade.

Among the agents that can induce brown fat differentiation from diverse lineages, members of the TGF-β superfamily play a compelling role. Members of this superfamily exhibit a dichotomous role as either inducers or inhibitors of adipogenesis. Specifically, myostatin (GDF-8) exhibits an anti-adipogenic activity while pharmacological inhibition of myostatin-activin receptor IIB interaction activates a functional brown adipogenic and thermogenic program [25]. A subclass of this superfamily, the BMPs (bone morphogenetic proteins) play vital role in regulating differentiation of diverse adipose depots. While BMP2 and BMP4 facilitate white adipocyte commitment and differentiation of multipotent mesenchymal C3H10T1/2 cells [26], BMP7 induces a robust brown fat specific gene program that drives favorable metabolic outcomes [27]. Most recently, through genetic ablation studies, the BMP receptor 1A (*BMPR1A*) has been implicated in *classical* brown fat differentiation from Myf5 positive lineage [28]. While these studies clearly identify the role for BMPs as drivers of adipocyte differentiation, it is imperative to assess the impact of direct BMP stimulation on muscle precursor cells in the context of *classical* brown adipogenesis.

In this study, we show that two days of BMP6 stimulation is sufficient to induce a significant brown fat differentiation response from C2C12 murine myoblast cells and human derived primary skeletal muscle precursor cells from multiple donors. Moreover, the response is stronger than that elicited by its closely related homologue, BMP7. Additional probing in C2C12 cells revealed that BMP6 does so in the absence of any exogenously forced gene expression and in the presence of insignificant levels of endogenous *Prdm16* mRNA. In an attempt to understand mechanisms underlying this favorable outcome, an unbiased transcriptional profiling array approach and subsequent gene knockdown studies identified the BMP6 target genes, Optineurin and Cyclooxygenase-2 as modulators of C2C12 myoblast to brown preadipocyte-like phenotype commitment and IGF-1R signaling as a key component of terminal differentiation of these BMP6 primed cells. Taken together, these data suggest that BMP6 might serve as a novel therapeutic that can enhance the amount of myogenic lineage derived brown fat.

## Materials and Methods

### Reagents

Recombinant human BMPs: BMP6 (507-BP-020), BMP7 (354-BP-010) and BMP8 (1073-BP-010) were purchased from R&D Systems. The following antibodies were purchased from Santa Cruz Biotechnology: Optn (sc-166576), Gapdh (sc-32233) and the following were purchased from Cell Signaling: Pparγ (2435S), Fabp4 (3544S), Adiponectin (2789S), and Cox2 (12282S). NS-398 (N194) was purchased from Sigma and dissolved in DMSO.

### Cell culture and lentiviral transductions

C2C12 cells were obtained from the American Type Culture Collection and maintained in Dulbecco's modified eagle medium (DMEM) supplemented with 10% FBS (Gibco) and 100 units/mL penicillin/streptomycin (Gibco). Cells were cultured at 37°C in a 5% CO_2_ humidified incubator. Primary human skeletal muscle precursor cells and the maintenance media were purchased from Lonza and ZenBio. The following shRNA lentiviral particles against relevant genes were purchased from Santa Cruz Biotechnology: Optn (sc-39055-V), Sdc3 (sc-41048-V), Smoc2 (sc-63047-V), Lgr6 (sc-60933-V), Prg4 (sc-60973-V), Cox2 (sc-29278-V), Igf-1 (sc-37194-V) and Igf-1Rα/β (sc-35638-V). Briefly, C2C12 cells plated in 24 well plates were infected with 150 μl of lentiviral particle supernatant (MOI = 3) in the presence of 6 μg/mL polybrene. After 48 hours, cells were transferred to 10 cm plates and maintained in selection media containing puromycin (1 μg/mL). Following death of mock infected cells, the stable pools of cells were used for the experiments and gene knockdown validated as described in Results.

### Adipocyte differentiation assay

8×10^4^ cells were cultured in maintenance media in the presence or absence of 250 ng/mL BMP for two days by the end of which, cells had attained confluency. Cells were then treated with induction hormone cocktail (0.5 mM isobutylmethylxanthine (IBMX), 125 nM indomethacin, 1 nM triiodothreonine (T3), 5 μM dexamethasone, 5 μg/mL insulin and 1 μM rosiglitazone) for three days. Subsequently cells were treated with post induction hormone cocktail (5 μg/mL insulin, 1 nM T3 and 1 μM rosiglitazone) for four days with post induction media replenished after two days. To induce thermogenesis, cells were stimulated with 10 μM forskolin for four hours. All reagents were purchased from Sigma unless noted otherwise.

### Oil-Red-O staining

#### For C2C12 derived adipocytes

Cells were washed once with PBS and fixed with 10% buffered formalin (Sigma) for 10 mins. Following a second wash with PBS, cells were incubated with staining solution for 15 mins. 0.5 g Oil-Red-O (Sigma) was dissolved in 100 mL isopropanol and 6 mL of this solution mixed with 4 mL distilled water (Gibco) was used as the staining solution. Cells were then rinsed with 60% isopropanol for 1 min, washed 2–3 times with distilled water and visualized. *For primary human skeletal muscle precursor cells derived adipocytes*: Cells were washed once with PBS and fixed with 10% buffered formalin for 30 mins. Following a wash with distilled water, cells were rinsed with 60% isopropanol for 5 mins. After aspirating isopropanol, Oil-Red-O staining solution (prepared as described above) was added to the cells. After 15–20 mins, cells were washed with distilled water and visualized. Bright field and Oil-Red-O images were captured using a Nikon DS-Fi2 camera mounted on a Nikon eclipseT*i* microscope and analyzed and quantified using NIS-elements BR 4.10.01 64-bit software.

### Immunofluorescence

**Figure 1:**
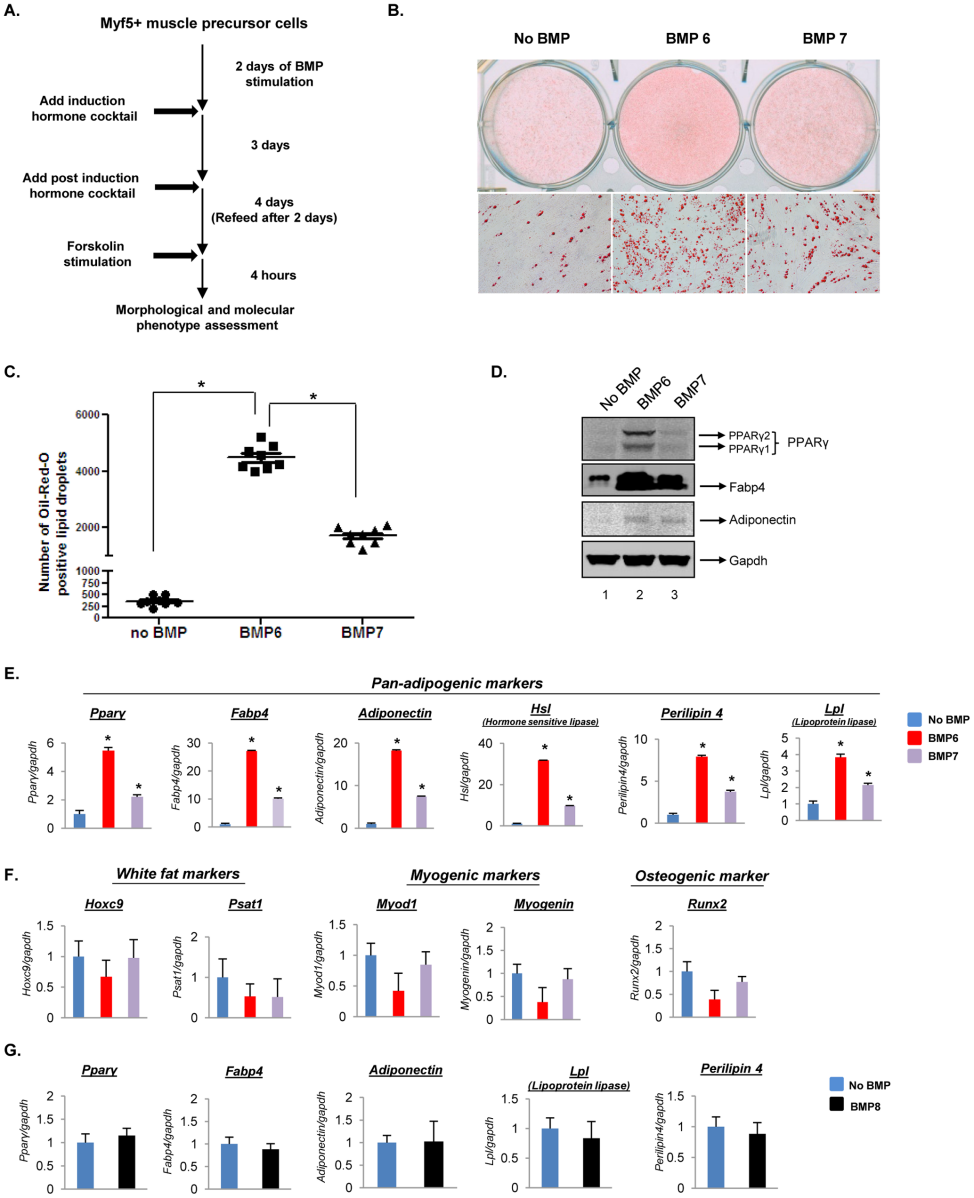
BMP6 drives a stronger adipogenic gene program in C2C12 muscle precursor cells compared to BMP7. (**A**) Schematic representation of the differentiation program indicating the time course of treatments. (**B**) Lipid accumulation was assessed by Oil-Red-O staining (magnification: 10× for second panel). (**C**) Quantification of Oil-Red-O positive lipid droplets for each condition from eight random microfields from three independent biological replicates. Error bars represent the standard error of the mean. (*p<0.05) (**D**) Immunoblot analyses for key pan-adipogenic markers, (**E**) Q-PCR analyses of pan-adipogenic markers (*Pparγ, Fabp4, Adiponectin, Hormone sensitive lipase, Perilipin 4, Lipoprotein lipase*), (**F**) white adipose tissue markers (*Hoxc9, Psat1*), myogenic markers (*Myod1, Myogenin*) and osteogenic marker (*Runx2*) in cells pretreated with or without BMP6 or BMP7 (250 ng/mL) followed by adipogenic differentiation. (**G**) Q-PCR analyses of pan-adipogenic markers in cells treated with or without 250 ng/mL BMP8 followed by adipogenic differentiation. Expression in untreated cells was set to 1 and results represent triplicate analyses of three independent biological replicates (mean ± SD), *p<0.05 versus no BMP. Similar results were obtained in at least 2–3 independent analyses.

2×10^4^ C2C12 cells were seeded in 96 well plates and stimulated with or without 250 ng/mL of BMP6 or BMP7. Following adipogenic differentiation described in Figure 1A, cells were fixed in 4% PFA for 30 mins at RT, washed and permeabilized with 0.5% Triton-X 100 for 20 mins at room temperature. After washing, cells were blocked in 2% normal donkey serum and subsequently stained with anti-Perilipin antibody (9349, Cell Signaling Technologies) used at 1:200 dilution overnight at 4°C. The following day cells were washed and stained with HCS CellMask Green, Hoechst 33342 and donkey anti-rabbit Alexa-Fluor 647 (Life Technologies). Images were acquired on a Leica SP5II laser scanning confocal microscope at 63× and 63× with 3× scan zoom with the pinhole set at 1 airy.

### Bioenergetics profile and Fatty acid oxidation assessment

Cellular bioenergetics profile of unstimulated and BMP stimulated C2C12 cells was determined using a Seahorse X24 Flux Analyzer. Briefly, 20×10^3^ C2C12 cells were seeded in 24 well Seahorse plates followed by two days of BMP stimulation (250 ng/mL) and adipogenic differentiation, as described in Figure 1A. Thereafter, OCR measurements were obtained at baseline and following injection of oligomycin (2 μM), carbonyl cyanide p-trifluoromethoxy phenylhydrazone (FCCP, 4 μM, Sigma) and rotenone (100 nM, Sigma) as described previously [29]. The calculations for basal respiration, mitochondrial proton leak and ATP turnover were determined as described in the Seahorse Operator's Manual. For fatty acid oxidation, the assay medium used was KRB buffer consisting of 110 mM NaCl, 4.7 mM KCl, 2 mM MgSO_4_, 1.2 mM Na_2_HPO_4_, 2.5 mM glucose adjusted to pH 7.4 supplemented with 0.5 mM carnitine. For induction of fatty acid oxidation, palmitate-BSA complex was injected to a final concentration of 250 μM. Fatty acid oxidation capacity was represented as increased OCR in response to palmitate-BSA complex compared to BSA only as control.

### Real time PCR and Immunoblot analyses

Total RNA from cultured cells was isolated using RNeasy Plus mini kit (Qiagen) and complementary DNA was synthesized from 1 μg of RNA using a high capacity cDNA reverse transcription kit (Applied Biosystems) according to the manufacturer's instructions. Taqman real time PCR analyses was performed in the Applied Biosystem's Vii A7 system. Quantification of transcript expression levels of relevant genes was performed using the ΔΔCt method. Pre-designed gene specific primers and probes were purchased from Applied Biosystems. For immunoblot analyses, cultured cells were lysed in RIPA buffer (Boston BioProducts) containing protease inhibitor cocktail (Roche). Following protein quantification using the BCA kit (Pierce), equal amounts of lysates were separated using SDS-PAGE (Novex) and transferred to nitrocellulose membranes (Invitrogen). Following one hour in blocking buffer (Odyssey) and overnight incubation with the relevant primary antibodies (at 4°C), membranes were incubated with secondary antibodies for 45 mins at room temperature. Development of membrane and quantification of band intensities were done using the Odyssey imaging system (LI-COR).

### Transcriptional profiling

Total RNA from cultured cells was purified using the RNeasy Plus Mini kit (Qiagen) as per manufacturer's recommendations. 300 ng of total RNA from each sample (3 independent biological replicates) was used to prepare biotinylated cRNA for analysis on the Mouse Genome 430 2.0 expression arrays (Affymetrix). Sample generation, processing, labeling and fragmenting was performed using the GeneChip 3′ IVT Express kit (Affymetrix). The GeneChip hybridization, wash and stain kit (Affymetrix) was used for sample hybridization to the mouse arrays and for array washing and staining using a Fluidics Station 450. Finally, arrays were scanned using a GeneChip Scanner 3000 7G with autoloader following the standard protocol. Gene expression profiling data was extracted from the Affymetrix Microarray Suite 5.0 (MAS 5.0) software and used for subsequent statistical analyses. Of the 45,101 probesets (qualifiers) on the microarray, 22,010 passed our primary quality assessment criteria. Probesets with an average expression value greater than 20 in any treatment group and a present ratio greater than 80% in any one group were included in the analysis. Transcripts were selected if their expression level was significantly different (*p≤0.05*, Student's *t* test) between untreated and treated samples (at matched time points), and if the fold change between the averaged expression value of the groups compared was greater than or equal to 1.5. Two way ANOVA was employed to select significantly changing genes (*p≤0.01*) between the treatment and time point compared. Statistically significant changing genes were represented by Eisen cluster display. In this format, each gene is represented by a single row and each sample, in triplicate, by a column. Two distinct clusters indicate genes induced (in red) or repressed (in green).

### Statistical assessment

Quantitative results are expressed as mean ± SD. Student's *t* test was used to calculate significance (*p≤0.05).

## Results

### BMP6 pretreatment converts C2C12 murine muscle precursor cells to adipocytes in conditions permissive for adipogenic differentiation

To investigate the effects of direct BMP6 stimulation on Myf5 positive myogenic cells, murine skeletal muscle derived C2C12 cells were utilized as a model system. These cells express salient features of myogenic lineage in that they are Myf5 proficient and inherently committed towards the myogenic differentiation pathway [30], [31]. Though C2C12 cells differentiate into well defined myotube like structures in pro-myogenic culture conditions, they retain the plasticity to differentiate into osteoblasts or brown adipocytes in a genetically engineered background [13], [23], [24], [32]. To investigate the molecular and morphological impact of BMP stimulation in the absence of forced gene expression, proliferating C2C12 cells were pretreated with or without 250 ng/mL BMP6 or BMP7 (a previously identified brown fat inducing morphogen [27]) followed by exposure to pro-adipogenic culture conditions as described in the Materials and Methods and illustrated in Figure 1A. 250 ng/mL dose was chosen based on adipogenic differentiation response achieved with different doses of BMP (Figure S1). As shown in Figure S2, unstimulated cells displayed poorly differentiated myotube like elongated structures following culture in pro-adipogenic media (*panels i and ii, first column*) indicating that these cells inherently progress towards myogenic differentiation pathway even in the presence of pro-adipogenic culture conditions. In contrast, the cells stimulated with BMP6 displayed a confluent fibroblast like morphology after 3 days in induction media that transitioned into lipid globule laden structures by the end of 4 days in post induction media (*second column*). Staining for cytoplasmic triglycerides using Oil-Red-O confirmed the identity of lipid globules, which were only minimally present in unstimulated cells (Figures 1B and 1C). A similar, yet significantly less potent response was observed in BMP7 stimulated cells where a heterogeneous confluent population of fibroblast cells and poorly differentiated myotube like elongated structures was evident by the end of 3 days in induction media followed by fewer lipid containing cells at the end of 4 days in post induction media (Figure S2, *third column*, Figures 1B and 1C). These morphological manifestations indicate that under the conditions used, BMP6 is a more potent inducer of adipogenesis than BMP7 in the Myf5 positive C2C12 cells.

**Figure 2:**
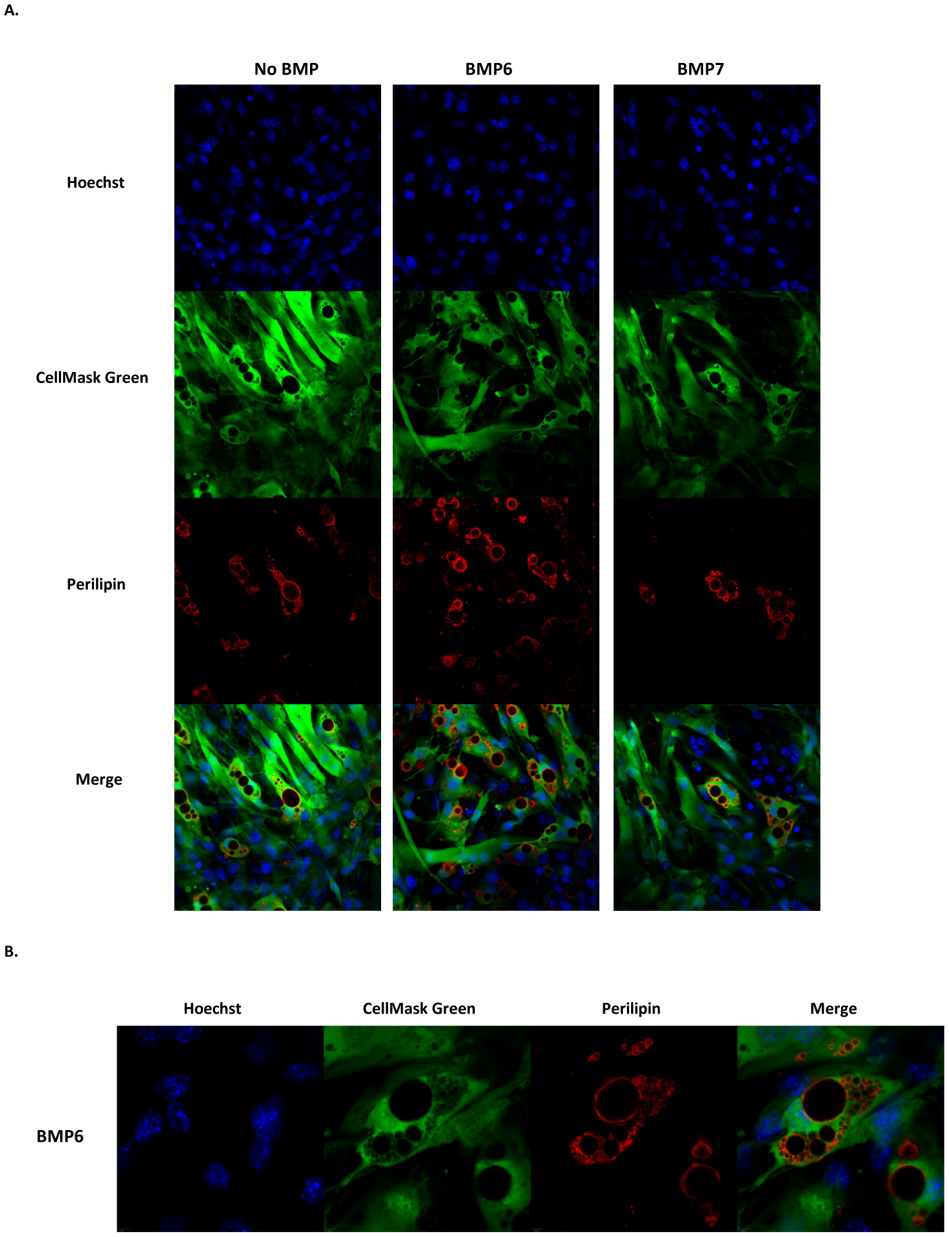
BMP6 programmed C2C12 cells exhibit abundance of lipid droplet tethered Perilipin. (**A**) Confocal images of C2C12 cells stimulated with or without BMP6 or BMP7 (250 ng/mL) for two days followed by adipogenic differentiation. Representative individual and merged images are shown for Hoechst (nuclear stain), CellMask Green (cytoplasmic stain) and Perilipin. (Magnification: 63X). (**B**) A region of BMP6 stimulated C2C12 derived adipocytes from panel A imaged at 3× scan zoom (Magnification: 63X) showing Perilipin surrounding multilocular lipid droplets.

Next, to ascertain the molecular identity of adipocytes generated via BMP pretreatment followed by culture in pro-adipogenic conditions, targeted protein expression and gene expression analyses were performed. As shown in Figure 1D, BMP6 stimulation caused significant induction in protein levels of pan-adipogenic markers including, PPARγ2, the main PPARγ isoform that drives adipogenesis [33], insulin sensitizing adipokine, Adiponectin [34] and Fabp4, a marker characteristic of differentiated adipocytes [35]. Assessment of this response at the transcript level confirmed the induction of pan-adipogenic gene expression elicited by BMP6 stimulation that was higher than that induced by BMP7 (Figure 1E). Confocal imaging revealed the abundance and cellular distribution of Perilipin, a marker for terminally differentiated adipocytes as being enriched in BMP6 reprogrammed C2C12 cells compared to unstimulated or BMP7 stimulated cells (Figure 2A). As expected, closer examination revealed that Perilipin is localized around the circumference of multilocular lipid droplets (Figure 2B). Interestingly, BMP6 and BMP7 stimulation did not induce white fat markers (*Hoxc9* and *Psat1*), myogenic markers (*Myod1 and Myogenin*) or the osteogenic marker (*Runx2*) (Figure 1F). BMP8, closely related to BMP6 and BMP7 [36], which was recently shown to increase brown fat thermogenic response [37], was then tested for its ability to induce adipogenesis. As shown in Figure 1G, two days of BMP8 pretreatment followed by culture in pro-adipogenic conditions did not induce adipocyte specific markers indicating that myogenic lineage derived adipogenic response is specific to BMP6 and BMP7. Collectively these data identify BMP6 and BMP7 as the novel adipocyte inducing morphogens in C2C12 myogenic cells, with BMP6 displaying a more potent response.

### BMP6 induces a significant brown fat gene program in C2C12 cells

**Figure 3:**
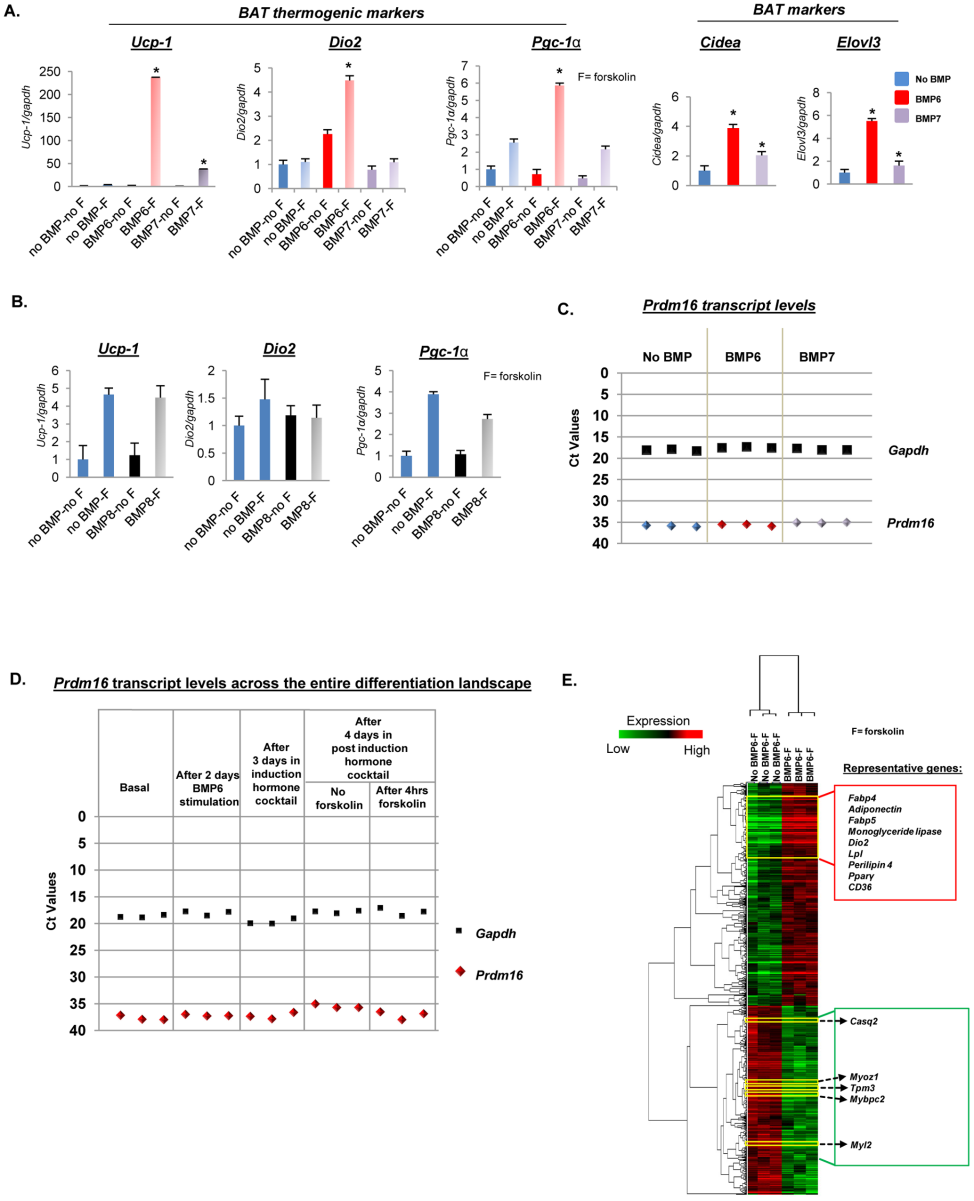
BMP6 induces a strong brown fat gene program in C2C12 cells without inducing *Prdm16.* (**A**) Q-PCR analyses of BAT thermogenic markers (*Ucp-1*, *Dio2* and *Pgc-1α*) and BAT specific markers (*Cidea* and *Elovl3*) in BMP6 or BMP7 (250 ng/mL) pretreated cells followed by adipogenic differentiation and stimulation with or without 10 μM forskolin for 4 hours. (**B**) Parallel analyses of BAT thermogenic markers in BMP8 (250 ng/mL) pretreated cells. The expression in untreated cells (no BMP pretreatment and no forskolin stimulation: no BMP-no F) was set to 1, and results represent triplicate analyses of three independent biological replicates (mean ± SD), *p<0.05 versus no BMP-F or no BMP, Student's *t* test. Similar results were obtained in at least two independent analyses. (**C**) Q-PCR generated Ct values for *Prdm16* and *Gapdh* transcripts at the end of differentiation cascade following BMP6 or BMP7 stimulation. (**D**) Ct values for *Prdm16* and *Gapdh* in relevant phases of differentiation landscape following BMP6 stimulation. Each dot per phase represents an independent biological replicate. For all the Q-PCR analyses, *Gapdh* served as the endogenous control. (**E**) Heat map depiction and unbiased clustering analyses of 1718 significantly changing qualifiers (*p≤0.05, fold change≥1.5*) in C2C12 cells stimulated with or without BMP6 for two days followed by differentiation protocol outlined in Figure 1A. Key brown fat and pan-adipogenic markers: *Fabp4, Adiponectin, Fabp5, Monoglyceride lipase, Dio2, Lipoprotein lipase (Lpl), Perilipin 4, Pparγ, and CD36,* and myogenic markers*: Calsequestrin 2 (Casq2), Myozenin 1 (Myoz1), Tropomyosin 3 (Tpm3), Myosin binding protein C-fast type (Mybpc2) and Myosin regulatory light chain 2 (Myl2)* are highlighted in the heat map. Each gene is represented by a single row and each sample in three independent biological replicates for each time point, by a column. Two distinct clusters indicate genes induced (red) and repressed (green), with representative induced and repressed genes highlighted in red and green boxes respectively.

To establish the brown fat molecular identity of BMP6 and BMP7 pretreated cells, transcript levels of BAT markers were measured following the differentiation cascade outlined in Figure 1A. As shown in Figure 3A, 250 ng/mL BMP6 treated cells displayed significant induction of brown fat specific genes: cAMP dependent thermogenic markers (*Ucp-1, Dio2* and *Pgc-1α*) and cAMP independent markers (*Cidea and Elovl3*), which were higher than in BMP7 stimulated cells. Concentrations of BMP6 below 250 ng/mL were ineffective at inducing robust *Ucp-1* levels (Figure S1B). The brown fat inducing response was specific to BMP6 and BMP7 as BMP8 failed to elicit a similar response (Figure 3B), consistent with its distal role in brown fat activation rather than differentiation [37]. Previous studies have identified a critical role for Prdm16 in driving the transition from skeletal muscle precursor cells to brown fat [12], [13]. To assess the expression levels of endogenous *Prdm16* in BMP6 and BMP7 reprogrammed C2C12 derived brown adipocytes, *Prdm16* transcript levels were measured (Figure 3C). Strikingly, the data revealed insignificant levels of *Prdm16* transcript (Ct values ≥ 35–40). To determine whether *Prdm16* was induced at any point during the differentiation protocol, thereby facilitating BMP6 driven brown adipogenesis, *Prdm16* transcript levels were measured at relevant time points. As shown in Figure 3D, there was no significant induction of *Prdm16* above the negligible levels observed at the basal state. These data are consistent with lack of significant endogenous *Prdm16* expression in C2C12 cells and primary murine myoblasts as reported previously [13], [23].

The above findings suggest a novel paradigm for brown fat differentiation from Myf5 positive muscle precursor cells as triggered through BMP6 stimulation. To confirm the molecular identity of the terminally differentiated cellular population obtained at the end of differentiation protocol on a global platform, a gene signature was generated from cells stimulated with or without BMP6 for two days followed by culture in pro-adipogenic conditions and stimulation with forskolin for 4 hours (as outlined in Figure 1A). As shown in Figure 3E, unsupervised clustering revealed two distinct clusters representing genes induced (red) or repressed (green) in BMP6 pretreated cells relative to the unstimulated cells. As expected, the induced gene cluster was primarily enriched in pan- and brown adipogenic genes (*Fabp4, Adiponectin, Fabp5, Monoglyceride lipase, Dio2, Lipoprotein lipase, Perilipin 4, Pparγ, and CD36,* also see Table S1 *)* whereas myogenic genes (*Calsequestrin 2, Myozenin 1, Tropomyosin 3, Myosin binding protein C-fast type and Myosin regulatory light chain 2)* were predominant in the repressed cluster. Collectively, these data highlight a novel and potent role for BMP6 in programming myogenic cells with negligible levels of endogenous *Prdm16* to a brown fat-like phenotype in pro-adipogenic culture conditions.

### BMP6 reprogrammed C2C12 cells exhibit a cellular bioenergetics phenotype characteristic of brown adipocytes

**Figure 4:**
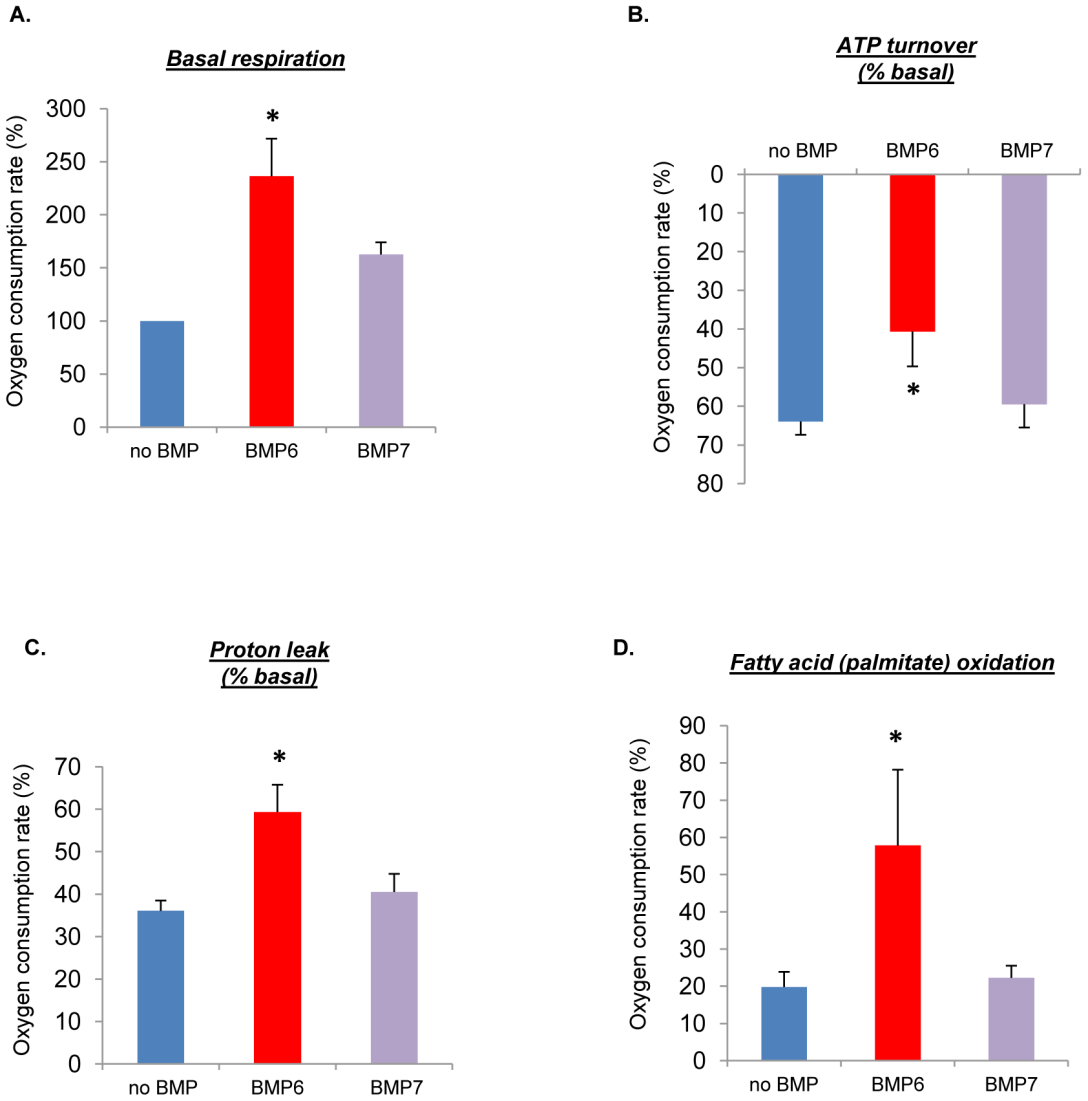
Adipocytes derived from C2C12 cells by BMP6 action exhibit bioenergetics phenotype characteristic of brown adipocytes. Oxygen consumption rate (OCR) measurements for (A) basal respiration, (B) ATP turn over, (C) proton leak and (D) fatty acid oxidation using a Seahorse XF24 extracellular flux analyzer following sequential administration of oligomycin, FCCP and rotenone. Results in (A) are plotted relative to unstimulated condition set to 100. Data in (B) and (C) are expressed as percent of basal respiration. Results represent cumulative analyses of multiple independent biological replicates from three independent experiments (n≥6 for each condition per experiment). (D) Fatty acid (palmitate) oxidation capacity is represented as increased OCR in response to palmitate-BSA complex compared to BSA only as control. Results represent cumulative analyses of multiple independent biological replicates from three independent experiments (n≥9 for palmitate-BSA and ≥9 for BSA for each condition, mean ± SD). *p<0.05 versus no BMP.

To complement the brown fat specific gene expression findings with downstream bioenergetics functional consequence, the oxygen consumption rates (OCR) for various components of mitochondrial respiration were measured using the Seahorse XF24 flux analyzer. As is shown in Figure 4A, BMP6 stimulated C2C12 cells exhibit significantly higher OCR for basal mitochondrial respiration than BMP7 stimulated cells supporting the finding that BMP6 is a more potent adipogenic morphogen than BMP7 in these cells (Figure 1 and Figure 2). OCR for ATP turnover and proton leak was then measured following administration of Oligomycin (F_1_F_0_ - ATP synthase inhibitor). BMP6 stimulated cells displayed 36.5% lower ATP linked OCR and 64.7% higher proton leak associated OCR compared to unstimulated cells (Figures 4B and 4C). These data demonstrate that BMP6 stimulated C2C12 derived adipocytes exhibit high uncoupling and OCR profile that is characteristic of brown adipocytes. Furthermore, as brown adipocytes utilize fatty acids as fuel to generate heat, fatty acid oxidation was subsequently measured in BMP6 or BMP7 reprogrammed cells. As shown in Figure 4D, OCR for exogenously added palmitic acid was increased significantly in BMP6 stimulated cells relative to unstimulated cells. BMP7 stimulated cells did not display significant differences in any of the measured parameters that is consistent with the lack of robust brown fat gene induction by BMP7 (Figure 3A). Collectively these data demonstrate that under the conditions used, adipocytes derived from C2C12 cells by BMP6 action display functional characteristics of brown adipocytes.

### BMP6 reprograms primary human skeletal muscle precursor cells into *Ucp-1* expressing adipocytes

**Figure 5:**
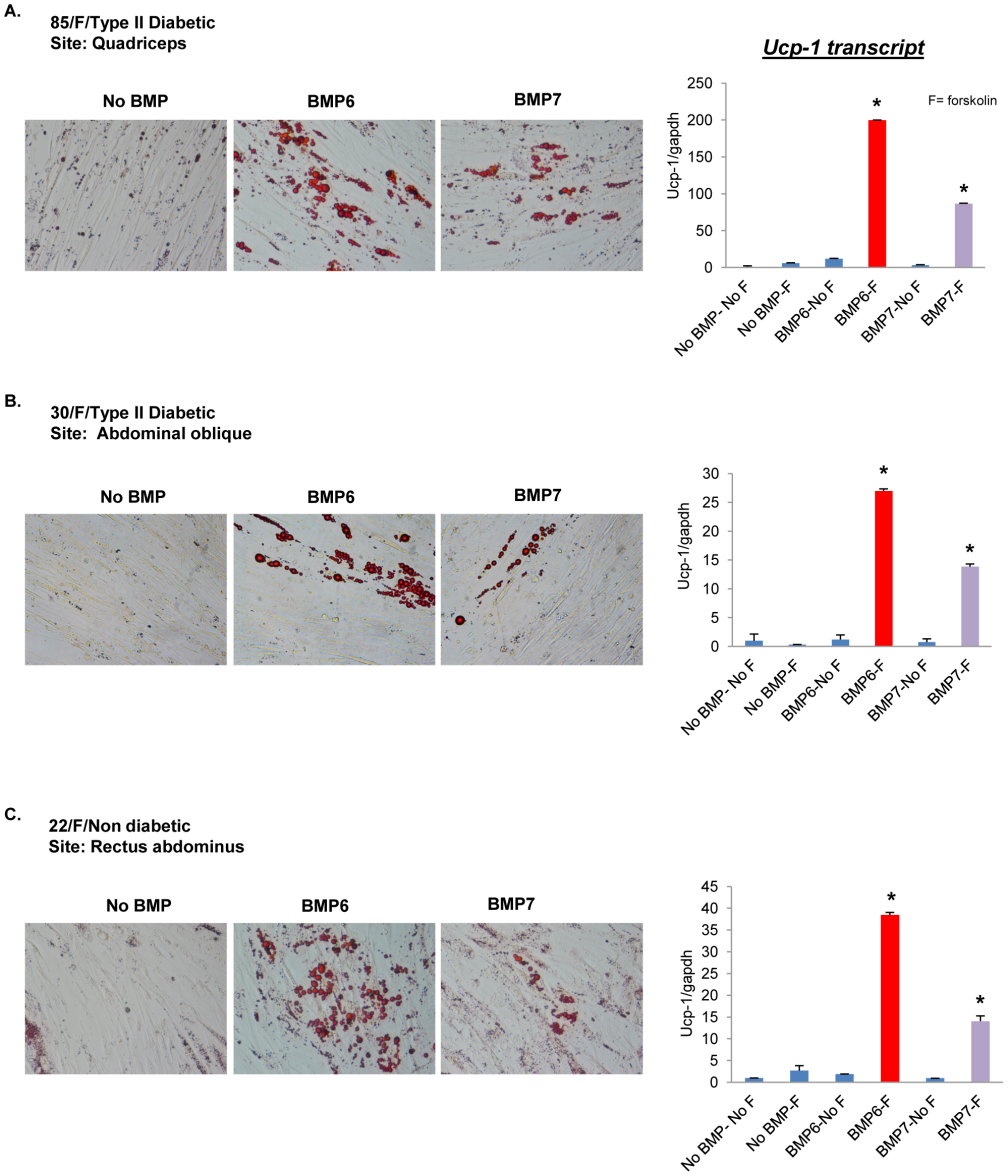
BMP6 induces lipid accumulation and *Ucp-1* expression in primary human skeletal muscle precursor cells from multiple donors. Primary human skeletal muscle precursor cells from type II diabetic or non diabetic donors isolated from distinct muscle sites were stimulated with or without BMP6 or BMP7 (250 ng/mL) for two days followed by adipogenic differentiation (as outlined in Figure 1A). Age/gender/diseased state for each donor and the site of myoblast isolation is specified. *Left panels*: Lipid accumulation in different donor cells was assessed by Oil-Red-O staining and a representative image is shown (magnification: 40X). *Right panels*: Q-PCR analyses of *Ucp-1* in BMP6 or BMP7 (250 ng/mL) pretreated cells followed by adipogenic differentiation and stimulation with or without 10 μM forskolin for 4 hours. The expression in untreated cells (no BMP pretreatment and no forskolin stimulation: no BMP-no F) was set to 1, and results represent triplicate analyses of three independent biological replicates (mean ± SD), *p<0.05 versus no BMP-F, Student's *t* test.

Given the dramatic effect of BMP6 stimulation in C2C12 cells and to confirm that the above described results were not a cell line specific response, the specificity of these findings was subsequently challenged in a clinically and physiologically relevant model system: primary human skeletal muscle precursor cells. To investigate the adipogenic effect of BMP6 and BMP7 on these cells, proliferating primary cells were stimulated with 250 ng/mL of BMP for two days followed by exposure to induction media and post induction media (scheme outlined in Figure 1A). As is shown in Figure 5A (*Left panel*), unstimulated cells differentiated into poor myotube like structures whereas BMP6 and BMP7 stimulated cells displayed lipid accumulation with the effect more pronounced upon BMP6 stimulation. Further probing to determine the molecular identity of these primary myoblasts derived adipocytes revealed that *Ucp-1* transcript was significantly induced (*Right panel*). These results were not donor specific as expansion of these analyses to two additional donor derived primary cells yielded similar results (Figures 5B and 5C). Consistent with data from C2C12 cells, the response in primary human cells was more potent with BMP6 compared to BMP7. Collectively these data demonstrate that BMP6 displays a novel brown fat inducing therapeutic potential not only in murine derived C2C12 cells but also in multiple human derived primary skeletal muscle precursor cells. While the follow up to observations in human derived cells is a subject of intense ongoing investigation in our laboratory, the subsequent analyses to elucidate the mechanism underlying this favorable BMP6 driven response were performed in C2C12 myoblast cells.

### BMP6 stimulation in C2C12 cells induces genes characteristic of preadipocytes

**Figure 6:**
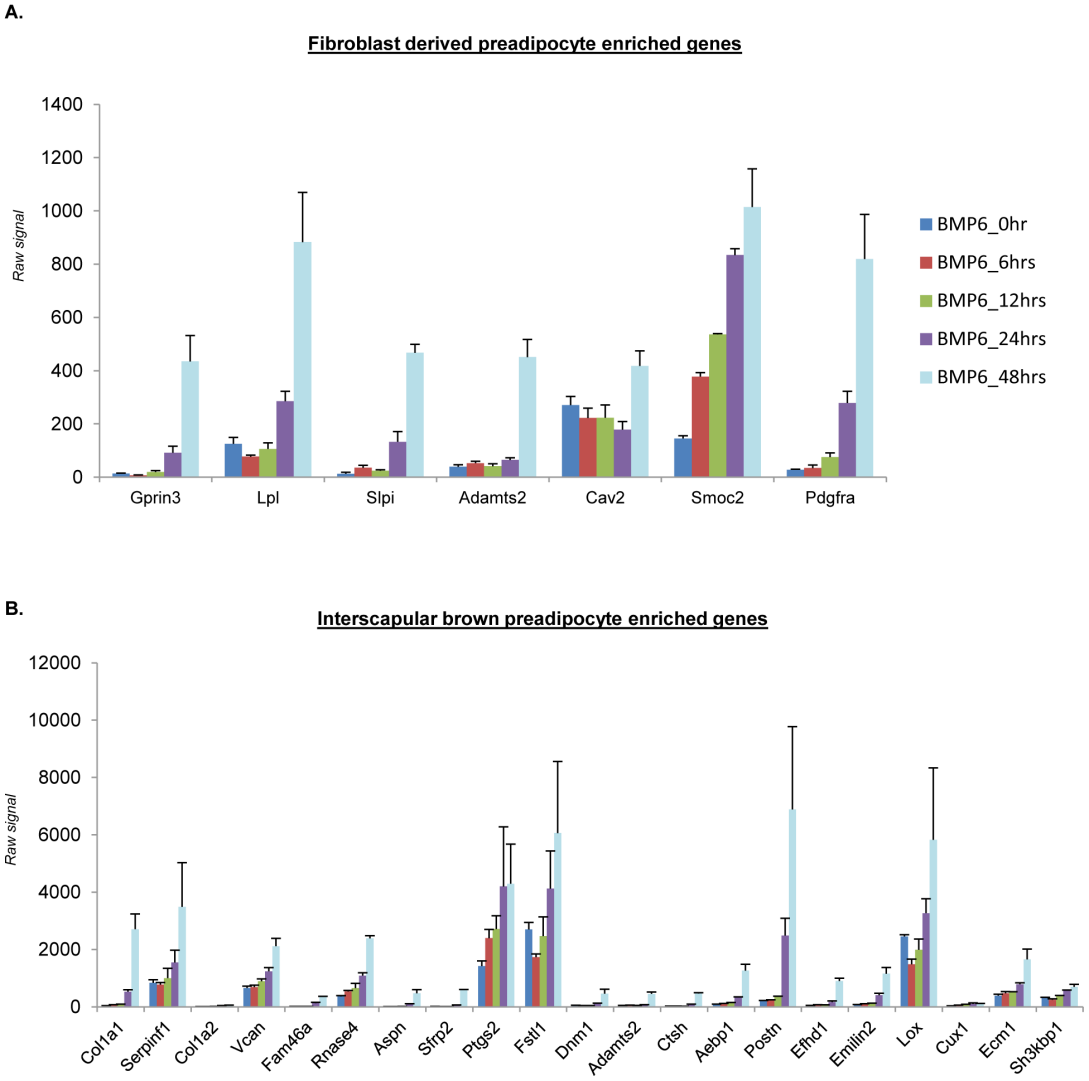
Two days of BMP6 stimulation in C2C12 cells induces genes characteristic of preadipocytes. Bar graph representation of genes characteristic of (**A**) fibroblast derived preadipocytes [39] and (**B**) murine interscapular brown preadipocytes [14], derived from Affymetrix array dataset generated from C2C12 cells stimulated with 250 ng/mL BMP6 and harvested at 0, 6, 12, 24 and 48 hours post BMP6 stimulation. Also see Figure 7B.

To better understand the mediators of BMP6 directed brown fat outcome, we performed global unbiased transcriptional profiling analyses at relevant time points in the differentiation cascade (strategy outlined in Figure S3). As preadipocytes exhibit a confluent fibroblast like morphology before entering terminal differentiation following culture in conditions permissive for adipogenesis [35], [38], we hypothesized that the confluent fibroblastic population achieved by the end of two days of BMP6 stimulation and before addition of induction media is enriched in brown preadipocyte-like cells. To validate this hypothesis, the expression levels of BMP6 induced genes were matched against markers representative of preadipocytes from two distinct published datasets. *First*, Gupta *et al*. identified markers characteristic of Swiss 3T3 fibroblast derived preadipocytes [39]. As shown in Figure 6A, few of those markers are BMP6 targets whose expression peak 48 hours post BMP6 stimulation indicating that the fibroblastic cellular population achieved at the end of two days of BMP stimulation is enriched in genes characteristic of preadipocytes. *Second*, Yin *et al*. identified mRNAs differentially expressed in murine brown preadipocytes isolated from interscapular BAT [14]. As shown in Figure 6B, brown preadipocyte enriched genes were induced following stimulation with BMP6. Collectively, these data demonstrate that BMP6 stimulated C2C12 cells exhibit induction of genes that are characteristic of fibroblast derived preadipocytes and murine interscapular brown preadipocytes and that the levels of these preadipocyte markers peak when cells had attained confluency (i.e. at 48 hours post BMP6 stimulation).

**Figure 7:**
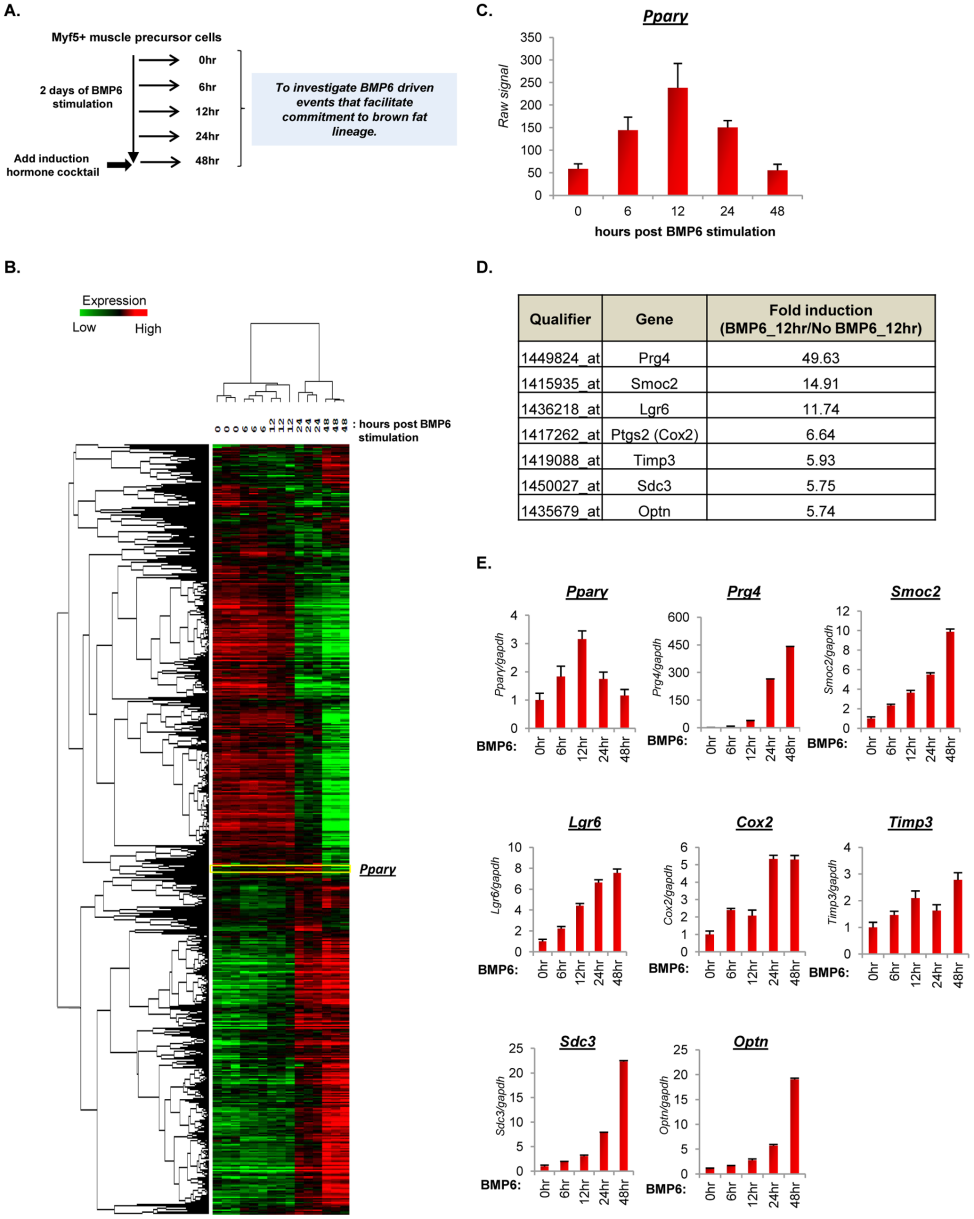
BMP6 pretreatment induces temporal expression of Pparγ and commitment phase candidate genes. (**A**) Schematic representation of the BMP6 pretreatment segment of differentiation landscape illustrated in Figure 1A indicating the time points at which cells were harvested. (**B**) Heat map depiction and unsupervised clustering analyses of BMP6 target gene signature in cells stimulated with 250 ng/mL BMP6 and harvested at 0, 6, 12, 24 and 48 hours post BMP6 stimulation. Two way ANOVA was employed to extract 4046 significantly changing qualifiers (*p≤0.01*) that were modulated as a function of treatment (BMP6 stimulation) and time (from 6 hours to 48 hours post BMP6 stimulation). *Pparγ* is highlighted in the heat map. Each gene is represented by a single row and each sample, in three independent biological replicates for each time point, by a column. Two distinct clusters indicate genes induced (red) and repressed (green). (**C**) Bar graph representation of *Pparγ* transcript levels at indicated time points measured using Affymetrix array. (**D**) Tabular representation of selected qualifiers, associated gene names and gene fold induction in cells stimulated with BMP6 relative to untreated cells at 12 hours. (**E**) Q-PCR validation of selected genes in cells stimulated with BMP6 for indicated time points. The expression in basal state (0 hour time point) was set to 1, and results represent triplicate analyses of three independent biological replicates (mean ± SD). Similar results were obtained in at least two independent analyses. Also see Figure S3.

Next, to identify the critical determinants of BMP6 driven myoblast to brown preadipocyte-like commitment response (Figure 7A), we generated a BMP6 target gene signature based on changes in gene expression resulting from treatment and time post BMP6 stimulation (Figure 7B). The unbiased clustering analyses revealed genes that were induced (red) or repressed (green) by BMP6 (also see Table S2). Interestingly, *Pparγ,* the master regulator of adipogenesis, exhibited a temporal expression pattern with transcript levels peaking at 12 hours and subsequently subsiding (Figure 7C). Pparγ is required for initiation, differentiation and maintenance of an adipocyte phenotype [40]. A recent study showed that the Zfp423 induced surge of Pparγ expression, primes preadipocytes to undergo differentiation when Pparγ levels cross a critical threshold following exposure to pro-adipogenic culture conditions [39]. Thus, we reasoned that the temporal peak expression of Pparγ serves as a commitment signal and we identified 12 hours post BMP6 stimulation as a crucial time point associated with programming of these cells to brown preadipocyte-like phenotype. To identify genes co-enriched at the 12 hour time point, we selected genes based on 2 criteria: (i) the candidate gene should have been previously implicated in physiological processes associated with adipogenic, myogenic, or brown fat differentiation and (ii) the gene should be induced more than 5 fold by BMP6 relative to levels observed in unstimulated cells (Figure 7D and Table S3). Genes that passed these criteria: *Prg4, Smoc2, Lgr6, Cox2, Timp3, Sdc3* and *Optn* [16], [41]–[49], were chosen for additional analyses. The transcript expression profiles of these genes were confirmed in an independent experiment and as shown in Figure 7E, their expression increased with levels peaking at 48 hours following BMP6 stimulation. Taken together, these results indicate that two days of BMP6 stimulation is sufficient to commit muscle precursor cells to preadipocyte-like phenotype driven in part by molecular events during a 12–48 hours period post BMP6 stimulation.

### Optineurin (Optn) and Cyclooxygenase-2 (Cox2) are involved in BMP6 induced brown fat differentiation

**Figure 8:**
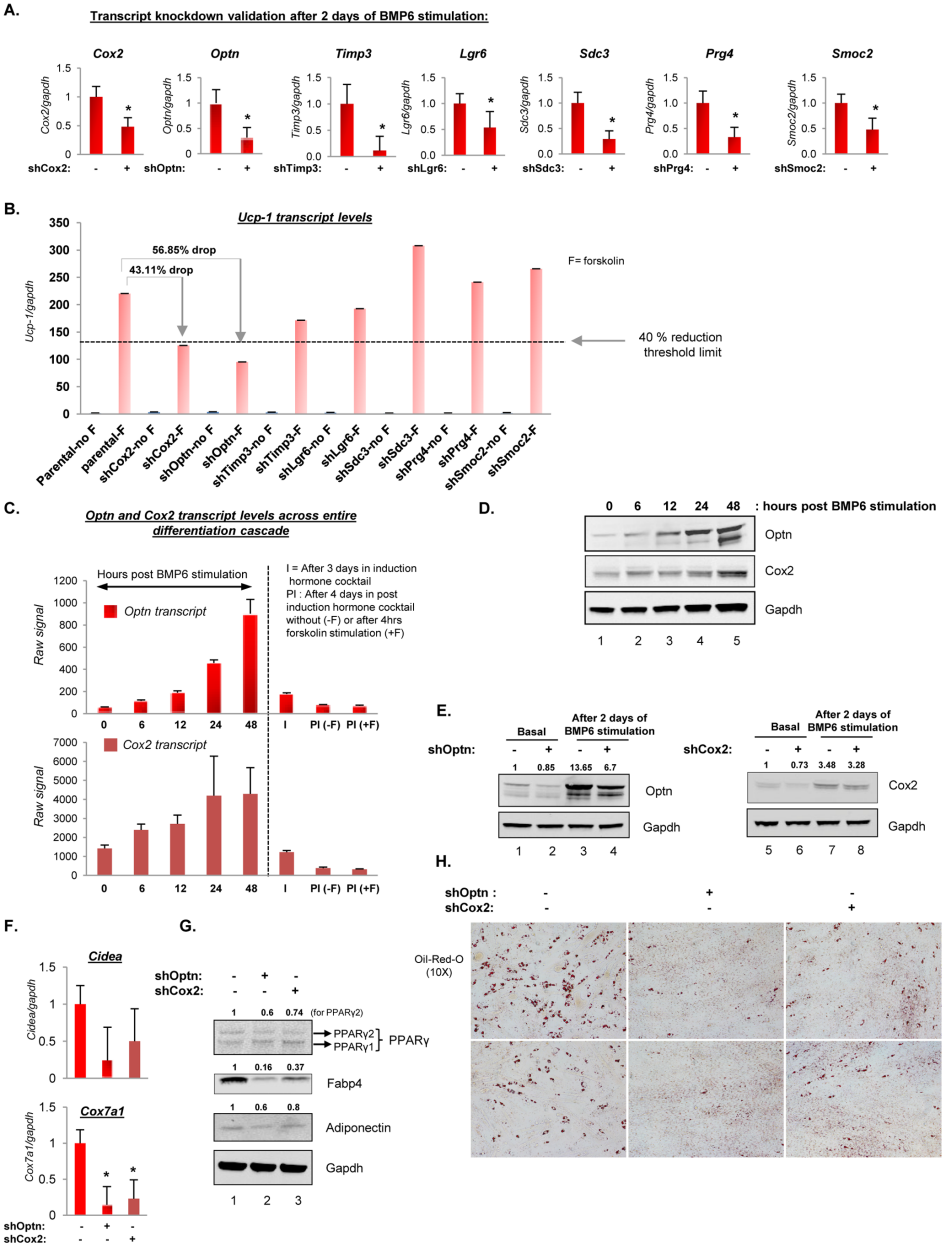
Knockdown of Optineurin (Optn) and Cyclooxygenase-2 (Cox2) impairs BMP6 induced brown fat differentiation. (**A**) Transcript knockdown validation of commitment phase candidate genes using Q-PCR (mean ± SD), *p<0.05. (**B**) Q-PCR validation for *Ucp-1* transcript levels in cells transduced with or without short hairpin constructs against indicated genes post two days BMP6 pretreatment followed by adipogenic differentiation and 10 uM forskolin stimulation. The expression in parental cells in the absence of forskolin stimulation was set to 1. A reduction in *Ucp-1* transcript levels by more than 40% of levels observed in forskolin stimulated parental cells was set as the lower threshold to choose the gene(s) of interest for subsequent investigations. Results represent triplicate analyses of two to three independent biological replicates (mean ± SD). (**C**) Bar graph representation of *Optn* and *Cox2* transcript levels at indicated time points measured using Affymetrix array. (**D**) Verification of Optn and Cox2 induction at the protein level by immunoblot analyses at indicated time points post BMP6 stimulation. Gapdh served as the loading control. (**E**) Knockdown of Optn and Cox2 was validated using immunoblot analyses in basal state and after two days of BMP6 stimulation. (**F**) Q-PCR validation for BAT markers (*Cidea, Cox7a1*) showing reduced induction in the context of attenuated Optn and Cox2 expression in indicated cells. Expression in *Optn* and *Cox2* proficient cells was set to 1 and *Gapdh* served as the endogenous control. Results represent triplicate analyses of three independent biological replicates and similar results were obtained in at least two independent analyses (mean ± SD), *p<0.05. (**G**) Immunoblot analyses for pan-adipogenic markers (PPARγ, Fabp4 and Adiponectin). Quantification of band intensity is provided above the respective blot. Note that the quantification data provided for PPARγ blot corresponds to the intensity of PPARγ2 (top band, panel G). (**H**) Two representative images for Oil-Red-O staining in Optn and Cox2 proficient and knockdown cells are shown. Analyses for panels F, G and H was performed at the end of the differentiation cascade illustrated in Figure 1A following two days of BMP6 (250 ng/mL) stimulation. Also see Figure S4 and Table S4.

To gain further insight into the molecular mechanisms mediating BMP6 induced commitment of C2C12 cells to brown fat lineage, the requirement of commitment phase candidate genes was assessed using knockdown studies. Model systems with stable candidate gene knockdown were generated using lentiviral particles (pool of at least 3 target specific shRNA constructs for each gene) and transcript knockdown validation was performed after two days of BMP6 stimulation. As shown in Figure 8A, significant knockdown was achieved for each candidate gene. Since *Ucp-1* is the clinical marker for heat generating brown fat and our data indicate that induction of *Ucp-1* is associated with high uncoupling in BMP6 reprogrammed C2C12 cells (Figure 4), *Ucp-1* transcript levels were utilized as the final readout for active brown fat differentiation. We set a 40% reduction of *Ucp-1* induction as a lower threshold and determined which candidate gene knock down affected this threshold. As shown in Figure 8B, cells with attenuated Optn and Cox2 expression displayed 56.85% and 43.11% drop in *Ucp-1* induction respectively, indicating that Optn and Cox2 knockdown is sufficient to attenuate BMP6 induced *Ucp-1* response below our set threshold limit. Hence, subsequent analyses were focused on understanding the role of Optn and Cox2 in mediating the BMP6 induced switch from myogenic to brown fat-like phenotype.

Optineurin (Optn) is a coiled coil containing multifunctional protein and mutations in *Optn* gene have been linked to amyotrophic lateral sclerosis and mitochondrial dysfunction in primary open angle glaucoma [49], [50]. Though it reportedly functions as a transcriptional modulator through interaction with Rab8, huntingtin, myosin phosphatase complex and transcription factor IIIA and as a negative regulator of NF-κB [51], it has no documented role(s) in adipogenesis. The second candidate gene, Cox2 (also known as Ptgs2, prostaglandin endoperoxide synthase 2) converts arachidonic acid to prostaglandins that are linked to proliferation, metastasis and angiogenesis. Cox2 over expression is associated with obesity induced inflammatory response [52] and most recently, Cox2 was implicated in control of energy homeostasis by recruitment of brown adipocytes in white fat [16], [53]. Although, Cox2 is enriched in murine interscapular brown preadipocytes [14], its role in *classical* brown adipogenesis is not clear. Since our findings link Optn and Cox2 knockdown to reduced *Ucp-1* induction, the consequence of Optn and Cox2 attenuation on adipocyte biology in the context of BMP6 stimulation was then assessed. As shown in Figure 8C, the *Optn* and *Cox2* transcript levels were not induced beyond 48 hours post BMP6 stimulation in the differentiation landscape, implying that Optn and Cox2 play a central role during the commitment phase. The induction of these two molecules was also manifested at the protein level (Figure 8D), demonstrating the cellular consequence of the observed induction in their transcript levels following BMP6 stimulation. The downstream implication of attenuated Optn and Cox2 expression (Figure 8E) on terminal differentiation was then evaluated. Following pretreatment with BMP6 for two days, Optn/Cox2 proficient, shOptn and shCox2 cells were cultured in pro-adipogenic conditions as outlined in Figure 1A, and molecular and morphological phenotypic assessment performed thereafter. As shown in Figure 8F, some (*Cidea* and *Cox7a1*) but not all (*Elovl3*, Figure S4A) were downregulated upon impairment of Optn and Cox2 expression. As expected, markers of terminally differentiated adipocytes were also attenuated in shOptn and shCox2 cells (Figure 8G and Table S4). The manifestation of these molecular events was also apparent at the morphological level as loss of Optn and Cox2 reduced the number of lipid containing cells as evidenced by decreased Oil-Red-O staining with results more pronounced in Optn attenuated setting (Figure 8H). To corroborate the results of Cox2 knockdown, Cox2 specific inhibitor NS-398 was employed [54]. NS-398 significantly impaired BMP6 induced adipogenic differentiation as evident by significantly lower lipid accumulation and pan adipogenic- and brown fat- marker expression, confirming that inhibition of Cox2 decreases the capacity of BMP6 to trigger myoblast to brown fat lineage commitment (Figure 4B onwards). Surprisingly, Cox2 specific inhibitor increased *Ucp-1* levels indicating a differential regulation of *Ucp-1* transcript. Collectively, these results indicate that under the conditions used, Optn and Cox2 modulate commitment of BMP6 stimulated C2C12 cells to brown fat lineage. To further explore the additional potential drivers of BMP6 induced commitment response and to place Optn and Cox2 in context of known adipogenic differentiation pathway(s), we employed Causal Reasoning Engine (See File S1 and Figure S5 and Table S5).

### IGF-1R drives terminal differentiation of BMP6 primed C2C12 cells

**Figure 9:**
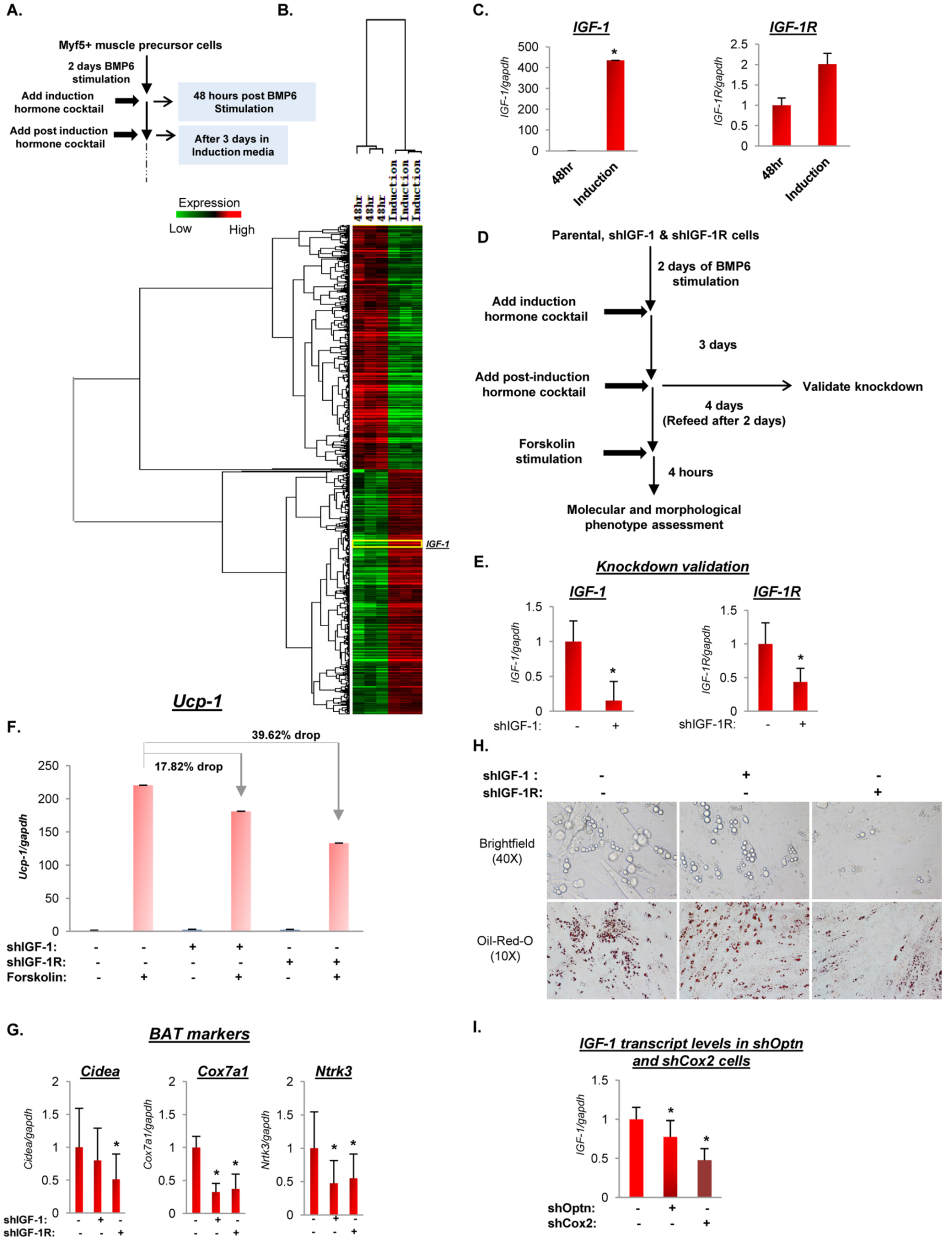
IGF-1R signaling drives terminal differentiation of BMP6 stimulated C2C12 cells in conditions permissive for adipogenic differentiation. (A) A schematic highlighting relevant time points in the differentiation protocol utilized to generate data shown in this figure. (B) Heat map depiction and unsupervised clustering analyses of 4255 significantly changing qualifiers (*p≤0.01, fold change≥1.5*) in BMP6 stimulated cells from “48 hours post BMP6 stimulation” to “post 3 days in induction media” time points. *IGF-1* levels are highlighted in the heat map. Each gene is represented by a single row and each sample in three independent biological replicates for each time point by a column. Two distinct clusters indicate genes induced (red) and repressed (green). (C) Independent Q-PCR validation of *IGF-1* and *IGF-1R* transcripts at indicated time points in the BMP6 stimulated cells. The expression at the 48 hour time point was set to 1, and results represent triplicate analyses of three independent biological replicates (mean ± SD), *p<0.05. (D) Schematic representation of the differentiation protocol for cells with or without *IGF-1* or *IGF-1R* knockdown is illustrated. (E) Knockdown validation of *IGF-1* and *IGF-1R* transcripts as indicated in panel D. The expression in the *IGF-1* or *IGF-1R* proficient cells was set to 1, and results represent triplicate analyses of three independent biological replicates (mean ± SD), *p<0.05. (F) Q-PCR validation for *Ucp-1* transcript levels in cells transduced with or without short hairpin constructs against indicated genes (IGF-1 or IGF-1R) post two days of BMP6 pretreatment followed by adipogenic differentiation and 4 hours forskolin stimulation. The expression in *IGF-1* and *IGF-1R* proficient cells in absence of forskolin stimulation was set to 1. (G) Q-PCR validation for BAT markers (*Cidea, Cox7a1, Ntrk3*) in indicated cells pretreated with BMP6 followed by adipogenic differentiation. Expression in *IGF-1* or *IGF-1R* proficient cells was set to 1 and *Gapdh* served as the endogenous control. Results represent triplicate analyses of three independent biological replicates (mean ± SD), *p<0.05. (H) Representative bright field images (magnification: 40X) and Oil-Red-O staining (magnification: 10X) in cells with attenuated IGF-1 or IGF-1R expression. (I) Q-PCR assessment of *IGF-1* transcript levels in Optn and Cox2 proficient and knockdown cells. Results represent triplicate analyses of three independent biological replicates (mean ± SD) and similar results were obtained in at least two independent analyses. Analyses for panels G and H was performed at the end of differentiation cascade illustrated in Figure 1A following two days of BMP6 (250 ng/mL) stimulation.

To identify the molecular network controlling terminal differentiation of BMP6 stimulated C2C12 cells that exhibit brown preadipocyte-like molecular phenotype, we created a gene signature between the “48 hours post BMP6 stimulation” time point and “following 3 days in induction media” (Figure 9A). As depicted in Figure 9B, the unsupervised clustering analyses revealed genes that were induced (red) or repressed (green) and identified *IGF-1* as one of the genes robustly induced after 3 days in induction media (also see Table S6). IGF-1 signaling has been previously implicated in adipogenic differentiation in different model systems [55], [56]. While the robust induction of *IGF-1* was verified in an independent experiment (Figure 9C, left panel), the levels of its cognate receptor, *IGF-1R* were modestly induced (Figure 9C, right panel). However, the lack of robust increase in *IGF-1R* does not exclude the possibility that basal levels (at 48 hour time point) are physiologically sufficient to contribute to events dictating terminal differentiation. This is consistent with high basal levels of IGF-1R observed in murine interscapular brown preadipocytes before the induction of differentiation [55], [57]. Though the components of IGF-1 signaling have previously been reported to mediate brown preadipocyte differentiation and brown fat thermogenic potential in altered genetic backgrounds [56], the implications of this pathway on differentiation of brown preadipocyte-like cells generated via BMP6 action have not been explored. To that end, we investigated the requirement of *IGF-1* and *IGF-1R* in differentiation of BMP6 primed cells through knockdown studies. Model systems of ligand or receptor stable knockdown were generated using lentiviral particles and attenuation was verified after 3 days in induction media (Figures 9D and 9E). The consequence of this event on active brown fat differentiation was then evaluated by measuring *Ucp-1* transcript levels. As shown in Figure 9F, attenuation of *IGF-1R* reduced *Ucp-1* induction by 39.6% compared to 17.8% in *IGF-1* impaired cells relative to levels achieved in IGF-1/IGF-1R proficient cells. Moreover, the abrogation of additional brown fat markers (*Cidea, Cox7a1 and Ntrk3*) was also observed (Figure 9G), indicating that the IGF-1/IGF-1R axis plays a vital role in terminal differentiation of BMP6 stimulated C2C12 cells in pro-adipogenic culture conditions. The consequence of these transcriptional events at a morphological level was assessed by monitoring the presence and accumulation of lipids. As shown in Figure 9H, only the *IGF-1R* knockdown cells exhibited fewer lipid globules and decreased lipid accumulation while *IGF-1* attenuated cells were indistinguishable from parental cells. These results indicate that the *IGF-1R* dependent events are vital for full molecular and morphological differentiation and maturation of brown preadipocyte-like cells. Given the sequential progression of events in the differentiation cascade and to corroborate the implications of Optn and Cox2 attenuation on ensuing terminal differentiation phase with Figure 8 results, *IGF-1* levels were measured in Optn and Cox2 knockdown cells following two days of BMP6 stimulation and three days culture in induction media. As expected, *IGF-1* levels were significantly reduced in shOptn and shCox2 cells (Figure 9I), thereby validating the involvement of Optn and Cox2 in mediating the myoblast to brown preadipocyte-like commitment switch. Collectively, these data indicate that *IGF-1R* signaling plays a pivotal role in terminal differentiation of brown preadipocyte-like cells derived from myoblast cells by BMP6 action.

## Discussion

The discovery of brown fat in adult humans presents an interesting therapeutic option in combating obesity and metabolic dysfunction [1]. Given the restricted amount of brown fat localized at discrete anatomical locations (cervical-supraclavicular, perirenal and paravertebral regions around major arteries), any options to enhance its amount and activity will provide novel therapeutic opportunities. The current study is the first to provide a potentially clinically relevant function of BMP6 in the context of myogenic lineage derived brown adipogenesis. Our data demonstrate that in the absence of any forced gene expression, BMP6 pretreatment is sufficient to program myogenic cells of murine and human origins into *Ucp-1* expressing adipocytes. Further investigation in C2C12 cells revealed that this BMP6 induced response is mediated in part by Optineurin and Cyclooxygenase-2. IGF-1R signaling then facilitates the terminal differentiation of BMP6 primed cells in pro-adipogenic culture conditions. The sequential contribution of these components in the differentiation cascade culminates in an active brown fat-like phenotype.

Identification of BMP6 as a novel brown fat inducing morphogen is significant as there is a scarcity of agents that can enhance the amount of myogenic lineage derived brown fat. The data herein demonstrate that the brown fat inducing therapeutic potential of BMP6 is not limited to murine derived C2C12 myoblast model system but also applicable to clinically relevant human derived primary skeletal muscle precursor cells. While our study is the first to show *in vitro* differentiation of primary human muscle precursor cells into *Ucp-1* expressing adipocytes in the absence of any forced gene expression, two groups have previously reported use of diverse human derived cellular model systems to generate brown adipocytes. Nishio *et al*. used a hematopoetin cocktail consisting of KIT ligand, fms-related tyrosine kinase 3 ligand, IL-6, VEGF and BMP7 to differentiate human embryonic stem cells and human inducible pluripotent stem cells into brown adipocytes [58] whereas Ahfeldt *et al*. reported programming of human pluripotent stem cell derived mesenchymal progenitor cells into brown adipocytes by ectopic expression of PPARγ2, C/EBP β and Prdm16 [59]. Despite these reports implicating human derived stem cells that may display high degree of lineage plasticity, our study highlights a novel and important role for BMP6 in lineage switch of myogenic committed human skeletal muscle precursor cells into *Ucp-1* expressing adipocytes. The mechanism by which BMP6 facilitates this favorable differentiation response and therapeutically relevant follow up studies to this novel observation remain an active area of investigation in our laboratory.

So far, at least four BMPs have been reported to facilitate different aspects of adipogenic differentiation. BMP2 and BMP4 drive mesenchymal lineage derived white adipogenesis whereas novel roles of BMP7 and BMP8 in brown adipocyte differentiation and activation have recently emerged [26], [27], [37]. BMPs function by binding to and activating serine/threonine kinase receptor complexes that are composed of type I and type II receptors. BMPs bind to five known type I receptors: Activin receptor like kinase 1 (ALK1), ALK2, ALK3 (also known as BMPR1A), ALK4 and ALK6 and three type II receptors: BMPR2, ActRIIa and ActRIIb. While incompletely understood, the binding specificity of BMPs to their receptors and the subsequent downstream signaling can be dictated by complex mechanisms including: (i) ligand and receptor concentrations, (ii) affinity of ligand to the receptor, (iii) inhibition of BMP action by BMP antagonist (e.g. noggin) that is induced following ligand stimulation. Although highly homologous, BMP6 and BMP7 bind the type I receptors with different specificities with BMP6 displaying twenty fold higher affinity for ALK3 than BMP7 [60]. In the context of brown adipogenic differentiation, this is of particular relevance as genetic ablation of ALK3 (BMPR1A) in Myf5 positive cells was recently shown to cause severe paucity of *classical* brown fat development [28]. This is in accordance with our data wherein BMP6 induced a stronger brown fat gene program compared to BMP7. Though the precise repertoire of receptors utilized by BMP6 in our model systems remains under investigation, it is intriguing to speculate that BMP6/BMPR1A interaction plays a pivotal role in inducing signaling mechanisms that conclude in genesis of *classical* brown fat. Moreover, the published study also reported compensatory browning of white fat in the absence of BMPR1A, indicating that the impact of BMPR1A signaling is not limited to Myf5 positive lineage but may also have favorable effects on the white fat. The effect of BMP6 stimulation on white fat in this context remains to be discerned. Nonetheless, these data collectively establish BMP signaling as a key pathway in brown fat development and activation, and introduce BMP6 as a potent inducer of myogenic lineage derived brown fat differentiation.

The requirement of Prdm16 in facilitating the myoblast to brown fat switch by interacting with pro-adipogenic transcription factors: PPARγ and C/EBP β is well established [12]. However, the absence of significant levels of endogenous *Prdm16* in our C2C12 myogenic model system at any point in the differentiation cascade revealed a novel facet of BMP6 induced brown fat differentiation process. Subsequent knockdown studies unmasked the requirement of Optn and Cox2 in promoting commitment of BMP6 stimulated C2C12 myoblasts to brown fat lineage. Optn is vital for cellular processes like membrane trafficking, protein secretion, cell division and host defense against pathogens driven in part through its interaction with a myriad of transcription factors (Rab8, Plk1, LC3, Myosin VI, Transferrin receptor) [51]. Cox2 is an important rate limiting enzyme in prostaglandin synthesis, which is involved in obesity associated inflammatory response and is expressed in murine interscapular brown preadipocytes [14], [52], [61]. Though Optn has previously not been implicated in fat differentiation or associated processes, one clinical study identified mitochondrial abnormalities in patients with primary open angle glaucoma, a pathological condition linked to mutations in Optn gene [49]. Another study has reported that a mutated form of Optn, E50K, identified in normal tension glaucoma patients, when overexpressed compromises the mitochondrial membrane potential in stress related conditions [62]. These data raise the interesting possibility that Optn dependent mechanisms could be involved in maintaining mitochondria biology and function in BMP6 primed C2C12 cells that differentiate into mitochondria rich, heat generating brown adipocytes. Cox2, on the other hand has previously been reported to facilitate or inhibit adipogenic differentiation in diverse model systems [63]–[68]. More recently, two independent groups published the role for Cox2 in browning of white fat with clinical implications in improving metabolic consequences of diet induced obesity [16], [53]. Interestingly, stimulation of C3H10T1/2 mesenchymal cells and primary human mesenchymal cells with prostaglandins during adipogenic differentiation generated *Ucp-1* expressing brown adipocytes [16] indicating that Cox2-prostaglandin signaling can drive brown fat differentiation from cell types that display lineage plasticity. Although the data herein provide an unanticipated and novel role for Optn in commitment of BMP6 stimulated C2C12 myoblast cells to a brown fat lineage, it also highlights the differential effects of Cox2 impairment. The apparent discrepancy in functional readout at the level of *Ucp-1* indicates towards diverse downstream mechanisms as induced by achieved knockdown versus drug inhibitor of Cox2. Collectively, these data put forth the hypothesis that while Cox2 modulates the myoblast to brown preadipocyte-like commitment, Cox2 activity also regulates *Ucp-1* gene transcription directly or indirectly. This notion is supported by the recent study from Madsen *et al.* who showed that Ucp-1 induction in white adipocytes is mediated by Cox2 activity [53]. It is also intriguing to speculate that the differential effect of these two modes of Cox2 inhibition in context of *Ucp-*1 is due to mutually exclusive target gene signatures that govern diverse events upstream of *Ucp-1*. In fact, Aryankalayil *et al.* have demonstrated that NS-398 and Cox2 RNAi yield significantly different gene expression profiles in tumor cells [69]. Thus further studies would be required to decipher the precise mechanisms underlying modulation of BMP6 driven adipogenic differentiation upon Optn and differential Cox2 impairment.

It is known that committed preadipocytes do not spontaneously undergo terminal differentiation in the absence of adipogenic stimuli. Upon addition of hormone cocktail, a transcriptional cascade is activated resulting in expression of metabolic genes and adipokines associated with mature fat cell phenotype [35]. Among other signaling pathways, Insulin/IGF-1 mediated signal transduction plays a key role in promoting terminal differentiation of brown preadipocytes [55], [56]. Given the higher expression levels of IGF-1R compared to insulin receptor in preadipocytes as reported previously [55], it is not surprising that impairment of IGF-1R would significantly inhibit the terminal differentiation of BMP6 primed C2C12 cells as observed herein. Moreover, both insulin and IGF-1 utilize IGF-1R as the primary receptor in preadipocytes to relay the downstream signals. Thus, the lack of substantial abrogation of brown fat thermogenic response and the absence of compromised morphological phenotype in IGF-1 knockdown cells in our studies could be explained in part by compensation for lack of IGF-1 by insulin, which is an important component of induction and post induction hormone cocktails. Published data and our results underscore an indispensible role for IGF-1R in initiating the signals that culminate in the induction of molecular and morphological brown adipocyte-like phenotype. While it was recently shown that fat specific insulin receptor and IGF-1 receptor knockout mice (FIGIRKO) display almost complete lack of interscapular brown fat explained in part through decreased C/EBP β phosphorlyation with resultant deregulation of C/EBP α and Pparγ [8], the expansion of these mechanistic findings have yet to be rigorously tested in the context of BMP6 stimulated cells. Collectively, these data reinforce the requirement of IGF-1R mediated signaling pathway in promoting differentiation of BMP6 stimulated myoblast cells or *de novo* brown preadipocytes (extracted from stromal vascular fraction of murine brown fat depot) in conditions permissive for adipogenesis.

One interesting perspective to be taken into consideration when attempting to place these findings in a physiological context is that though BMP6 reprogrammed C2C12 cells show significant brown fat specific molecular, morphological and bioenergetics phenotype, primal physiological differences do exist between cells that have been ectopically reprogrammed to brown fat like cells and endogenous brown adipocytes (as found *in vivo*). Of note, levels of *Elovl3* are significantly lower in BMP6 reprogrammed C2C12 cells compared to levels observed in murine interscapular BAT (data not shown) indicating that BMP6 reprogrammed cells, while significantly advance towards brown adipocyte fate, do show significant quantitative differences from native endogenous brown adipocytes. Not surprisingly, levels of other brown fat specific markers may also significantly differ in cells that have been reprogrammed by BMP6 to brown fat lineage via *in vitro* differentiation protocol versus endogenous brown adipocytes that are generated from inherently programmed precursor cells in an *in vivo* milieu.

In summary, the findings reported herein present a novel role for BMP6 in inducing brown fat differentiation from murine and primary human skeletal muscle precursor cells in the absence of any ectopic gene expression. Moreover these data identify Optineurin, Cyclooxygenase-2 and IGF-1R as endogenous regulators of BMP6 driven brown fat differentiation in C2C12 myoblast cells. Taken together, the present study identifies a new brown fat inducing role for BMP6 in cell based model systems. It also lays the foundation for future studies to test the potential use of BMP6 for brown fat differentiation in preclinical *in vivo* model systems directed towards effective obesity and body weight management.

## Supporting Information

Figure S1 Dose dependent response of BMP stimulation at driving adipogenic differentiation in C2C12 cells.
(TIF)

Figure S2 BMP6 stimulated C2C12 cells display morphology indicative of non myogenic lineage commitment.
(TIF)

Figure S3 Schematic of experimental strategy for transcriptional profiling study.
(TIF)

Figure S4 Achieved Cox2 and Optn knockdown does not diminish BMP6 induced *Elovl3* levels and differential effects of Cox2 selective inhibitor NS-398 on BMP6 induced brown fat differentiation in C2C12 cells.
(TIF)

Figure S5 Causal Reasoning Engine (CRE) identifies potential causal drivers of BMP6 programmed C2C12 myoblast to brown preadipocyte-like switch.
(TIF)

File S1 Supporting information containing text for Figure S5, Table S5 and figure legends for all the supplementary figures.
(DOC)

Table S1 Associated with Figure 3E.
(XLS)

Table S2 Associated with Figure 7B.
(XLS)

Table S3 Associated with Figure 7D.
(XLS)

Table S4 Associated with Figure 8G.
(XLS)

Table S5 Associated with Figure S5.
(XLS)

Table S6 Associated with Figure 9B.
(XLS)

## References

[1] Cypess AM, Lehman S, Williams G, Tal I, Rodman D, et al (2009) Identification and importance of brown adipose tissue in adult humans. N Engl J Med 360: 1509–1517. 19357406 10.1056/NEJMoa0810780 PMC2859951

[2] van Marken Lichtenbelt WD, Vanhommerig JW, Smulders NM, Drossaerts JM, Kemerink GJ, et al (2009) Cold-activated brown adipose tissue in healthy men. N Engl J Med 360: 1500–1508. 19357405 10.1056/NEJMoa0808718

[3] Ouellet V, Labbe SM, Blondin DP, Phoenix S, Guerin B, et al (2012) Brown adipose tissue oxidative metabolism contributes to energy expenditure during acute cold exposure in humans. J Clin Invest 122: 545–552. 22269323 10.1172/JCI60433 PMC3266793

[4] Virtanen KA, Lidell ME, Orava J, Heglind M, Westergren R, et al (2009) Functional brown adipose tissue in healthy adults. N Engl J Med 360: 1518–1525. 19357407 10.1056/NEJMoa0808949

[5] Cypess AM, White AP, Vernochet C, Schulz TJ, Xue R, et al.. (2013) Anatomical localization, gene expression profiling and functional characterization of adult human neck brown fat. Nat Med. 10.1038/nm.3112 PMC3650129 23603815

[6] Lidell ME, Betz MJ, Leinhard OD, Heglind M, Elander L, et al.. (2013) Evidence for two types of brown adipose tissue in humans. Nat Med. 10.1038/nm.3017 23603813

[7] Orava J, Nuutila P, Lidell ME, Oikonen V, Noponen T, et al (2011) Different metabolic responses of human brown adipose tissue to activation by cold and insulin. Cell Metab 14: 272–279. 21803297 10.1016/j.cmet.2011.06.012

[8] Stanford KI, Middelbeek RJ, Townsend KL, An D, Nygaard EB, et al (2013) Brown adipose tissue regulates glucose homeostasis and insulin sensitivity. J Clin Invest 123: 215–223. 23221344 10.1172/JCI62308 PMC3533266

[9] Bartelt A, Bruns OT, Reimer R, Hohenberg H, Ittrich H, et al (2011) Brown adipose tissue activity controls triglyceride clearance. Nat Med 17: 200–205. 21258337 10.1038/nm.2297

[10] Timmons JA, Wennmalm K, Larsson O, Walden TB, Lassmann T, et al (2007) Myogenic gene expression signature establishes that brown and white adipocytes originate from distinct cell lineages. Proc Natl Acad Sci U S A 104: 4401–4406. 17360536 10.1073/pnas.0610615104 PMC1810328

[11] Sanchez-Gurmaches J, Hung CM, Sparks CA, Tang Y, Li H, et al (2012) PTEN loss in the Myf5 lineage redistributes body fat and reveals subsets of white adipocytes that arise from Myf5 precursors. Cell Metab 16: 348–362. 22940198 10.1016/j.cmet.2012.08.003 PMC3488151

[12] Kajimura S, Seale P, Kubota K, Lunsford E, Frangioni JV, et al (2009) Initiation of myoblast to brown fat switch by a PRDM16-C/EBP-beta transcriptional complex. Nature 460: 1154–1158. 19641492 10.1038/nature08262 PMC2754867

[13] Seale P, Bjork B, Yang W, Kajimura S, Chin S, et al (2008) PRDM16 controls a brown fat/skeletal muscle switch. Nature 454: 961–967. 18719582 10.1038/nature07182 PMC2583329

[14] Yin H, Pasut A, Soleimani VD, Bentzinger CF, Antoun G, et al (2013) MicroRNA-133 controls brown adipose determination in skeletal muscle satellite cells by targeting Prdm16. Cell Metab 17: 210–224. 23395168 10.1016/j.cmet.2013.01.004 PMC3641657

[15] Wu J, Bostrom P, Sparks LM, Ye L, Choi JH, et al (2012) Beige adipocytes are a distinct type of thermogenic fat cell in mouse and human. Cell 150: 366–376. 22796012 10.1016/j.cell.2012.05.016 PMC3402601

[16] Vegiopoulos A, Muller-Decker K, Strzoda D, Schmitt I, Chichelnitskiy E, et al (2010) Cyclooxygenase-2 controls energy homeostasis in mice by de novo recruitment of brown adipocytes. Science 328: 1158–1161. 20448152 10.1126/science.1186034

[17] Kim KH, Jeong YT, Oh H, Kim SH, Cho JM, et al (2013) Autophagy deficiency leads to protection from obesity and insulin resistance by inducing Fgf21 as a mitokine. Nat Med 19: 83–92. 23202295 10.1038/nm.3014

[18] Li P, Fan W, Xu J, Lu M, Yamamoto H, et al (2011) Adipocyte NCoR knockout decreases PPARgamma phosphorylation and enhances PPARgamma activity and insulin sensitivity. Cell 147: 815–826. 22078880 10.1016/j.cell.2011.09.050 PMC3783197

[19] Qiang L, Wang L, Kon N, Zhao W, Lee S, et al (2012) Brown remodeling of white adipose tissue by SirT1-dependent deacetylation of Ppargamma. Cell 150: 620–632. 22863012 10.1016/j.cell.2012.06.027 PMC3413172

[20] Schulz TJ, Tseng YH (2013) Brown adipose tissue: development, metabolism and beyond. Biochem J 453: 167–178. 23805974 10.1042/BJ20130457 PMC3887508

[21] Hu E, Tontonoz P, Spiegelman BM (1995) Transdifferentiation of myoblasts by the adipogenic transcription factors PPAR gamma and C/EBP alpha. Proc Natl Acad Sci U S A 92: 9856–9860. 7568232 10.1073/pnas.92.21.9856 PMC40901

[22] Mancini A, El Bounkari O, Norrenbrock AF, Scherr M, Schaefer D, et al (2007) FMIP controls the adipocyte lineage commitment of C2C12 cells by downmodulation of C/EBP alpha. Oncogene 26: 1020–1027. 16909111 10.1038/sj.onc.1209853

[23] Sun L, Xie H, Mori MA, Alexander R, Yuan B, et al (2011) Mir193b-365 is essential for brown fat differentiation. Nat Cell Biol 13: 958–965. 21743466 10.1038/ncb2286 PMC3149720

[24] Rajakumari S, Wu J, Ishibashi J, Lim HW, Giang AH, et al.. (2013) EBF2 Determines and Maintains Brown Adipocyte Identity. Cell Metab. 10.1016/j.cmet.2013.01.015 PMC3622114 23499423

[25] Fournier B, Murray B, Gutzwiller S, Marcaletti S, Marcellin D, et al (2012) Blockade of the activin receptor IIb activates functional brown adipogenesis and thermogenesis by inducing mitochondrial oxidative metabolism. Mol Cell Biol 32: 2871–2879. 22586266 10.1128/MCB.06575-11 PMC3416189

[26] Huang H, Song TJ, Li X, Hu L, He Q, et al (2009) BMP signaling pathway is required for commitment of C3H10T1/2 pluripotent stem cells to the adipocyte lineage. Proc Natl Acad Sci U S A 106: 12670–12675. 19620713 10.1073/pnas.0906266106 PMC2722335

[27] Tseng YH, Kokkotou E, Schulz TJ, Huang TL, Winnay JN, et al (2008) New role of bone morphogenetic protein 7 in brown adipogenesis and energy expenditure. Nature 454: 1000–1004. 18719589 10.1038/nature07221 PMC2745972

[28] Schulz TJ, Huang P, Huang TL, Xue R, McDougall LE, et al (2013) Brown-fat paucity due to impaired BMP signalling induces compensatory browning of white fat. Nature 495: 379–383. 23485971 10.1038/nature11943 PMC3623555

[29] Vernochet C, Mourier A, Bezy O, Macotela Y, Boucher J, et al (2012) Adipose-specific deletion of TFAM increases mitochondrial oxidation and protects mice against obesity and insulin resistance. Cell Metab 16: 765–776. 23168219 10.1016/j.cmet.2012.10.016 PMC3529641

[30] Beauchamp JR, Heslop L, Yu DS, Tajbakhsh S, Kelly RG, et al (2000) Expression of CD34 and Myf5 defines the majority of quiescent adult skeletal muscle satellite cells. J Cell Biol 151: 1221–1234. 11121437 10.1083/jcb.151.6.1221 PMC2190588

[31] Lindon C, Montarras D, Pinset C (1998) Cell cycle-regulated expression of the muscle determination factor Myf5 in proliferating myoblasts. J Cell Biol 140: 111–118. 9425159 10.1083/jcb.140.1.111 PMC2132595

[32] Fux C, Mitta B, Kramer BP, Fussenegger M (2004) Dual-regulated expression of C/EBP-alpha and BMP-2 enables differential differentiation of C2C12 cells into adipocytes and osteoblasts. Nucleic Acids Res 32: e1. 14704358 10.1093/nar/gnh001 PMC373304

[33] Tontonoz P, Hu E, Spiegelman BM (1994) Stimulation of adipogenesis in fibroblasts by PPAR gamma 2, a lipid-activated transcription factor. Cell 79: 1147–1156. 8001151 10.1016/0092-8674(94)90006-x

[34] Yamauchi T, Kamon J, Waki H, Terauchi Y, Kubota N, et al (2001) The fat-derived hormone adiponectin reverses insulin resistance associated with both lipoatrophy and obesity. Nat Med 7: 941–946. 11479627 10.1038/90984

[35] Cristancho AG, Lazar MA (2011) Forming functional fat: a growing understanding of adipocyte differentiation. Nat Rev Mol Cell Biol 12: 722–734. 21952300 10.1038/nrm3198 PMC7171550

[36] Bragdon B, Moseychuk O, Saldanha S, King D, Julian J, et al (2011) Bone morphogenetic proteins: a critical review. Cell Signal 23: 609–620. 20959140 10.1016/j.cellsig.2010.10.003

[37] Whittle AJ, Carobbio S, Martins L, Slawik M, Hondares E, et al (2012) BMP8B increases brown adipose tissue thermogenesis through both central and peripheral actions. Cell 149: 871–885. 22579288 10.1016/j.cell.2012.02.066 PMC3383997

[38] Cristancho AG, Schupp M, Lefterova MI, Cao S, Cohen DM, et al (2011) Repressor transcription factor 7-like 1 promotes adipogenic competency in precursor cells. Proc Natl Acad Sci U S A 108: 16271–16276. 21914845 10.1073/pnas.1109409108 PMC3182685

[39] Gupta RK, Arany Z, Seale P, Mepani RJ, Ye L, et al (2010) Transcriptional control of preadipocyte determination by Zfp423. Nature 464: 619–623. 20200519 10.1038/nature08816 PMC2845731

[40] Rosen ED, MacDougald OA (2006) Adipocyte differentiation from the inside out. Nat Rev Mol Cell Biol 7: 885–896. 17139329 10.1038/nrm2066

[41] Chang Y, She ZG, Sakimura K, Roberts A, Kucharova K, et al (2012) Ablation of NG2 proteoglycan leads to deficits in brown fat function and to adult onset obesity. PLoS One 7: e30637. 22295099 10.1371/journal.pone.0030637 PMC3266271

[42] Liu P, Pazin DE, Merson RR, Albrecht KH, Vaziri C (2009) The developmentally-regulated Smoc2 gene is repressed by Aryl-hydrocarbon receptor (Ahr) signaling. Gene 433: 72–80. 19146932 10.1016/j.gene.2008.12.010 PMC2652666

[43] Alexander DL, Ganem LG, Fernandez-Salguero P, Gonzalez F, Jefcoate CR (1998) Aryl-hydrocarbon receptor is an inhibitory regulator of lipid synthesis and of commitment to adipogenesis. J Cell Sci 111 (Pt 22): 3311–3322. 9788873 10.1242/jcs.111.22.3311

[44] Strader AD, Reizes O, Woods SC, Benoit SC, Seeley RJ (2004) Mice lacking the syndecan-3 gene are resistant to diet-induced obesity. J Clin Invest 114: 1354–1360. 15520868 10.1172/JCI20631 PMC524223

[45] Fuentealba L, Carey DJ, Brandan E (1999) Antisense inhibition of syndecan-3 expression during skeletal muscle differentiation accelerates myogenesis through a basic fibroblast growth factor-dependent mechanism. J Biol Chem 274: 37876–37884. 10608853 10.1074/jbc.274.53.37876

[46] Bernot D, Barruet E, Poggi M, Bonardo B, Alessi MC, et al (2010) Down-regulation of tissue inhibitor of metalloproteinase-3 (TIMP-3) expression is necessary for adipocyte differentiation. J Biol Chem 285: 6508–6514. 20056610 10.1074/jbc.M109.078444 PMC2825446

[47] Meng Q, Lv J, Ge H, Zhang L, Xue F, et al (2012) Overexpressed mutant optineurin(E50K) induces retinal ganglion cells apoptosis via the mitochondrial pathway. Mol Biol Rep 39: 5867–5873. 22422156 10.1007/s11033-011-1397-7

[48] Nath M, Offers M, Hummel M, Seissler J (2011) Isolation and in vitro expansion of Lgr6-positive multipotent hair follicle stem cells. Cell Tissue Res 344: 435–444. 21484413 10.1007/s00441-011-1165-y

[49] Abu-Amero KK, Morales J, Bosley TM (2006) Mitochondrial abnormalities in patients with primary open-angle glaucoma. Invest Ophthalmol Vis Sci 47: 2533–2541. 16723467 10.1167/iovs.05-1639

[50] Maruyama H, Kawakami H (2012) Optineurin and amyotrophic lateral sclerosis. Geriatr Gerontol Int. 10.1111/ggi.12022 23279185

[51] Kachaner D, Genin P, Laplantine E, Weil R (2012) Toward an integrative view of Optineurin functions. Cell Cycle 11: 2808–2818. 22801549 10.4161/cc.20946

[52] Subbaramaiah K, Morris PG, Zhou XK, Morrow M, Du B, et al (2012) Increased levels of COX-2 and prostaglandin E2 contribute to elevated aromatase expression in inflamed breast tissue of obese women. Cancer Discov 2: 356–365. 22576212 10.1158/2159-8290.CD-11-0241 PMC3398487

[53] Madsen L, Pedersen LM, Lillefosse HH, Fjaere E, Bronstad I, et al (2010) UCP1 induction during recruitment of brown adipocytes in white adipose tissue is dependent on cyclooxygenase activity. PLoS One 5: e11391. 20613988 10.1371/journal.pone.0011391 PMC2894971

[54] Futaki N, Takahashi S, Yokoyama M, Arai I, Higuchi S, et al (1994) NS-398, a new anti-inflammatory agent, selectively inhibits prostaglandin G/H synthase/cyclooxygenase (COX-2) activity in vitro. Prostaglandins 47: 55–59. 8140262 10.1016/0090-6980(94)90074-4

[55] Boucher J, Tseng YH, Kahn CR (2010) Insulin and insulin-like growth factor-1 receptors act as ligand-specific amplitude modulators of a common pathway regulating gene transcription. J Biol Chem 285: 17235–17245. 20360006 10.1074/jbc.M110.118620 PMC2878077

[56] Boucher J, Mori MA, Lee KY, Smyth G, Liew CW, et al (2012) Impaired thermogenesis and adipose tissue development in mice with fat-specific disruption of insulin and IGF-1 signalling. Nat Commun 3: 902. 22692545 10.1038/ncomms1905 PMC3529640

[57] Entingh-Pearsall A, Kahn CR (2004) Differential roles of the insulin and insulin-like growth factor-I (IGF-I) receptors in response to insulin and IGF-I. J Biol Chem 279: 38016–38024. 15247278 10.1074/jbc.M313201200

[58] Nishio M, Yoneshiro T, Nakahara M, Suzuki S, Saeki K, et al (2012) Production of functional classical brown adipocytes from human pluripotent stem cells using specific hemopoietin cocktail without gene transfer. Cell Metab 16: 394–406. 22958922 10.1016/j.cmet.2012.08.001

[59] Ahfeldt T, Schinzel RT, Lee YK, Hendrickson D, Kaplan A, et al (2012) Programming human pluripotent stem cells into white and brown adipocytes. Nat Cell Biol 14: 209–219. 22246346 10.1038/ncb2411 PMC3385947

[60] Vukicevic S, Grgurevic L (2009) BMP-6 and mesenchymal stem cell differentiation. Cytokine Growth Factor Rev 20: 441–448. 19900832 10.1016/j.cytogfr.2009.10.020

[61] Hsieh PS, Jin JS, Chiang CF, Chan PC, Chen CH, et al (2009) COX-2-mediated inflammation in fat is crucial for obesity-linked insulin resistance and fatty liver. Obesity (Silver Spring) 17: 1150–1157. 19247274 10.1038/oby.2008.674

[62] De Marco N, Buono M, Troise F, Diez-Roux G (2006) Optineurin increases cell survival and translocates to the nucleus in a Rab8-dependent manner upon an apoptotic stimulus. J Biol Chem 281: 16147–16156. 16569640 10.1074/jbc.M601467200

[63] Kim DH, Puri N, Sodhi K, Falck JR, Abraham NG, et al (2013) Cyclooxygenase-2 dependent metabolism of 20-HETE increases adiposity and adipocyte enlargement in mesenchymal stem cell-derived adipocytes. J Lipid Res 54: 786–793. 23293373 10.1194/jlr.M033894 PMC3617952

[64] Ghoshal S, Trivedi DB, Graf GA, Loftin CD (2011) Cyclooxygenase-2 deficiency attenuates adipose tissue differentiation and inflammation in mice. J Biol Chem 286: 889–898. 20961858 10.1074/jbc.M110.139139 PMC3013048

[65] Fajas L, Miard S, Briggs MR, Auwerx J (2003) Selective cyclo-oxygenase-2 inhibitors impair adipocyte differentiation through inhibition of the clonal expansion phase. J Lipid Res 44: 1652–1659. 12837847 10.1194/jlr.M300248-JLR200

[66] Yan H, Kermouni A, Abdel-Hafez M, Lau DC (2003) Role of cyclooxygenases COX-1 and COX-2 in modulating adipogenesis in 3T3-L1 cells. J Lipid Res 44: 424–429. 12576525 10.1194/jlr.M200357-JLR200

[67] Bell-Parikh LC, Ide T, Lawson JA, McNamara P, Reilly M, et al (2003) Biosynthesis of 15-deoxy-delta12,14-PGJ2 and the ligation of PPARgamma. J Clin Invest 112: 945–955. 12975479 10.1172/JCI18012 PMC193665

[68] Petersen RK, Jorgensen C, Rustan AC, Froyland L, Muller-Decker K, et al (2003) Arachidonic acid-dependent inhibition of adipocyte differentiation requires PKA activity and is associated with sustained expression of cyclooxygenases. J Lipid Res 44: 2320–2330. 12923227 10.1194/jlr.M300192-JLR200

[69] John-Aryankalayil M, Palayoor ST, Cerna D, Falduto MT, Magnuson SR, et al (2009) NS-398, ibuprofen, and cyclooxygenase-2 RNA interference produce significantly different gene expression profiles in prostate cancer cells. Mol Cancer Ther 8: 261–273. 19139136 10.1158/1535-7163.MCT-08-0928 PMC2861287

