# Supplementary figures and images for: Brown Fat Determination and Development from Muscle Precursor Cells by Novel Action of Bone Morphogenetic Protein 6
Source: PLoS One. 2014 Mar 21;9(3):e92608. doi: 10.1371/journal.pone.0092608 (PMC3962431; doi:10.1371/journal.pone.0092608)

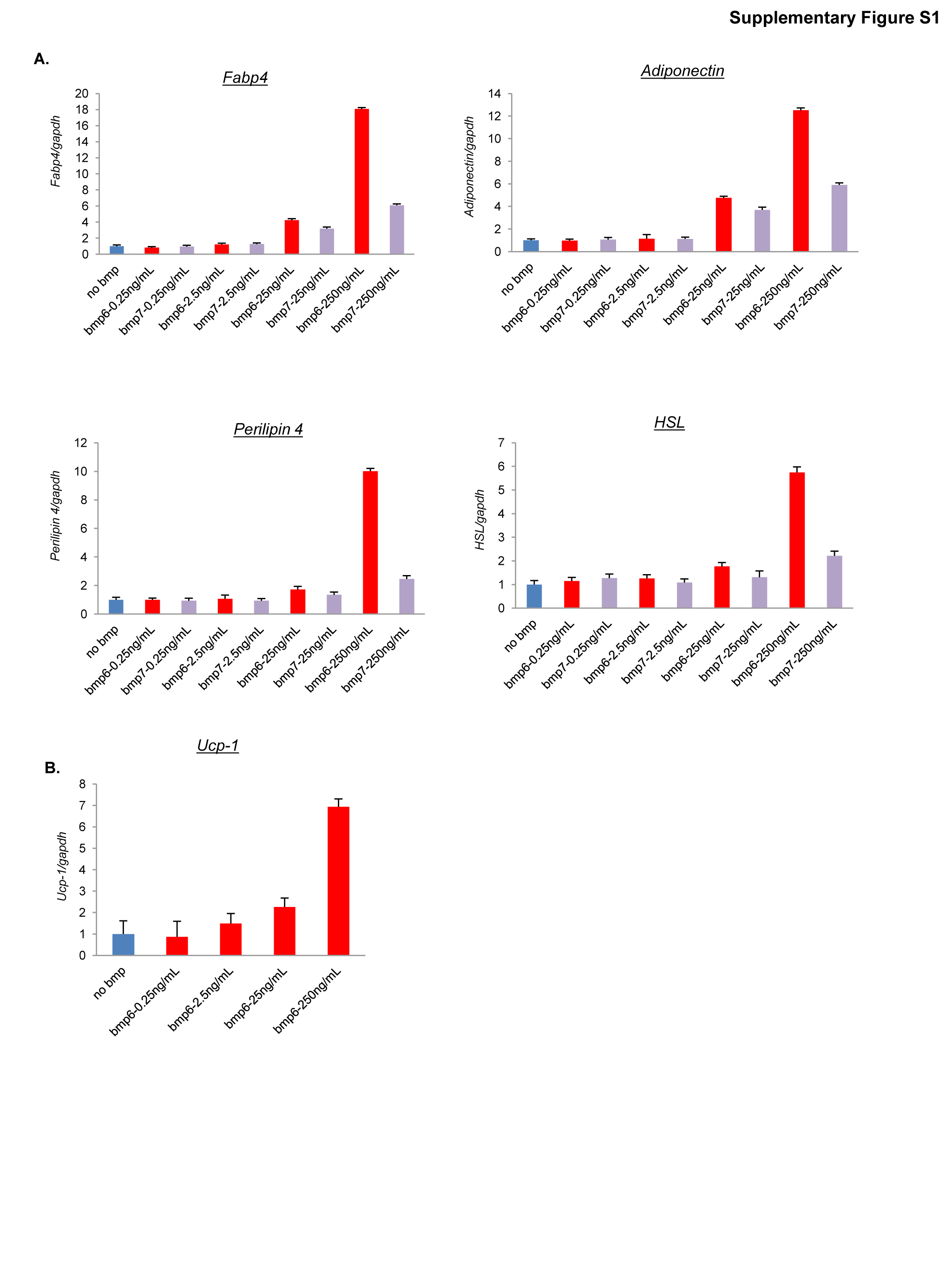

Supplement: Figure S1 — Dose dependent response of BMP stimulation at driving adipogenic differentiation in C2C12 cells. (TIF) [file pone.0092608.s001.tif]

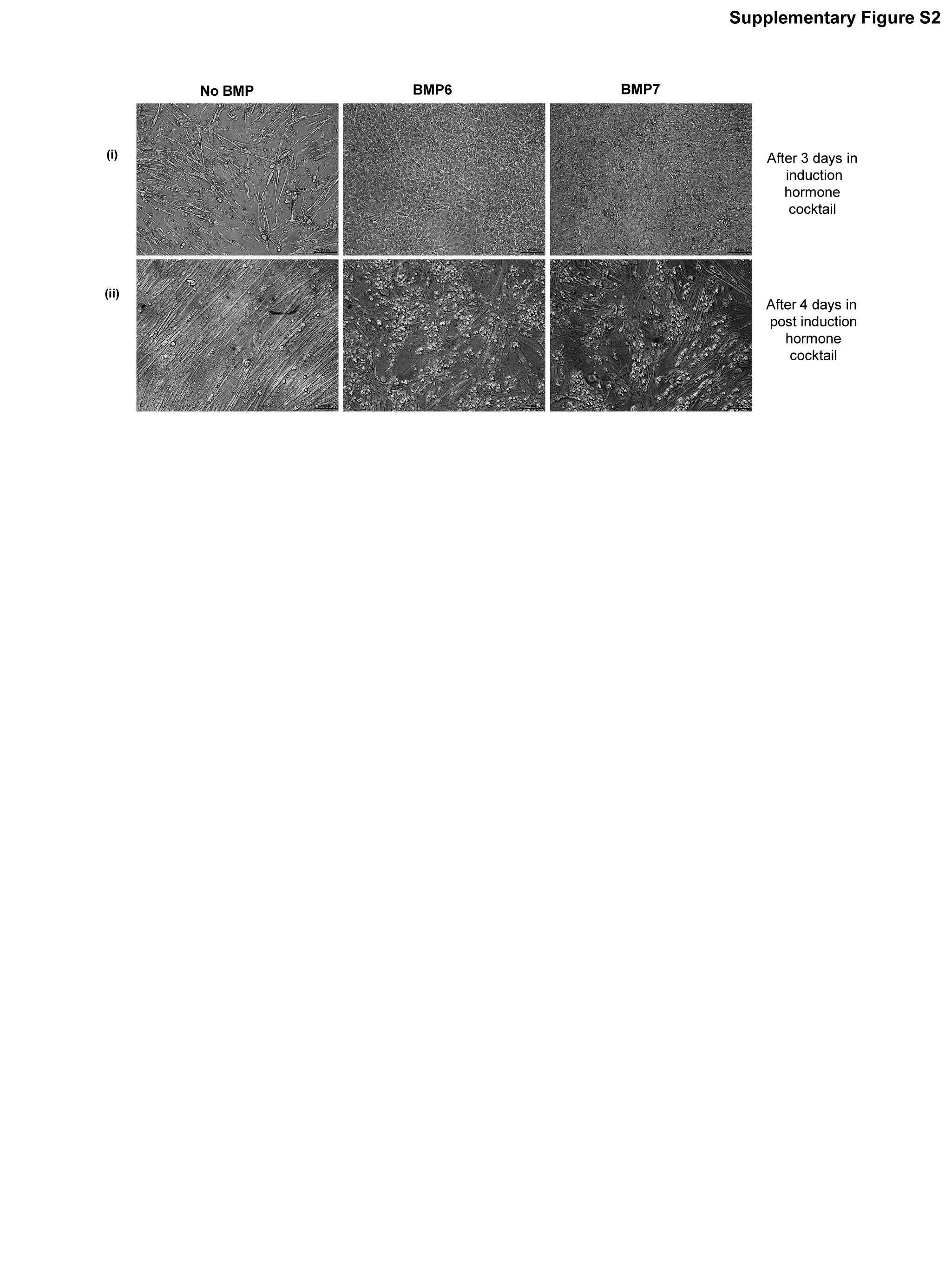

Supplement: Figure S2 — BMP6 stimulated C2C12 cells display morphology indicative of non myogenic lineage commitment. (TIF) [file pone.0092608.s002.tif]

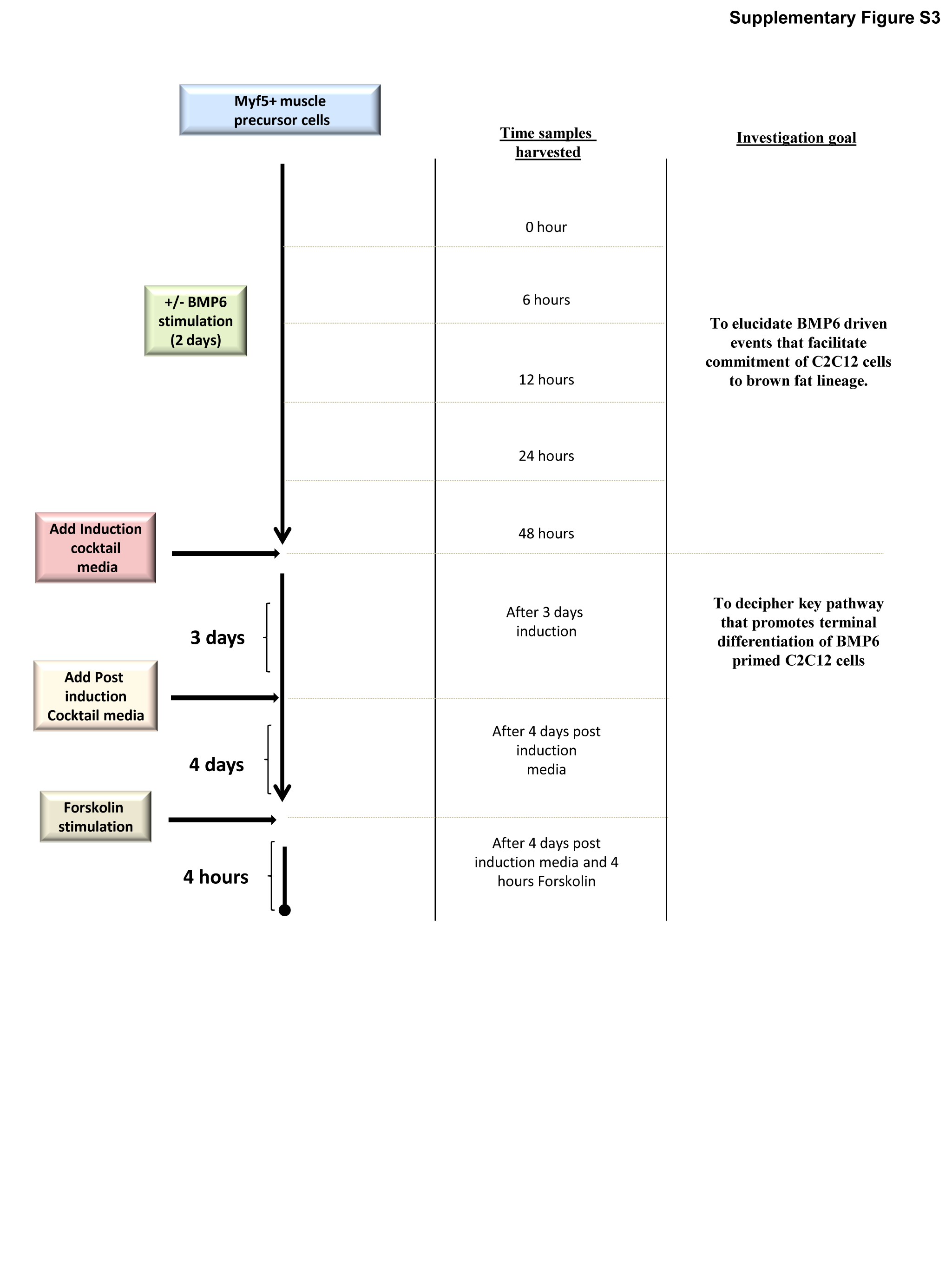

Supplement: Figure S3 — Schematic of experimental strategy for transcriptional profiling study. (TIF) [file pone.0092608.s003.tif]

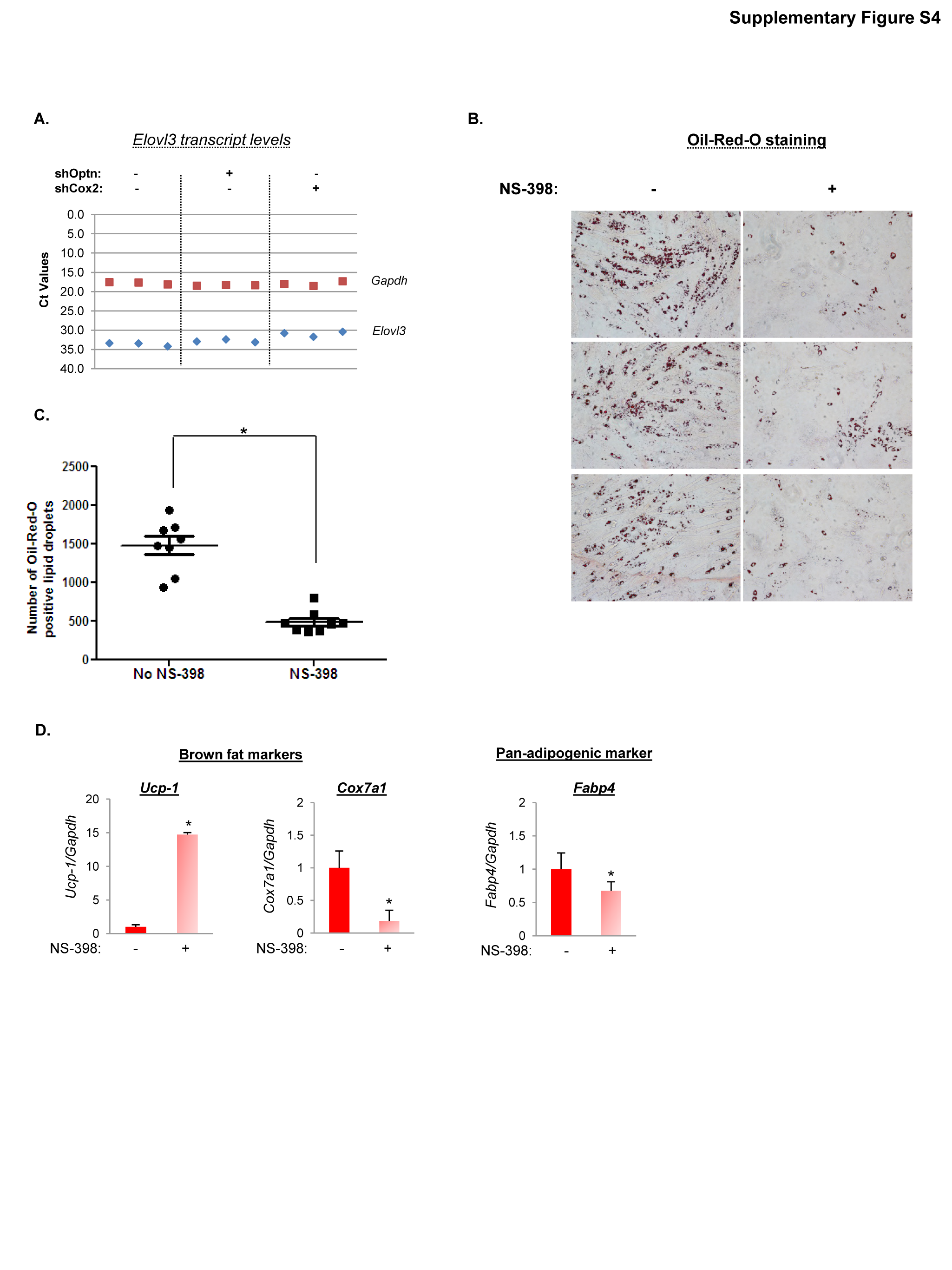

Supplement: Figure S4 — Achieved Cox2 and Optn knockdown does not diminish BMP6 induced Elovl3 levels and differential effects of Cox2 selective inhibitor NS-398 on BMP6 induced brown fat differentiation in C2C12 cells. (TIF) [file pone.0092608.s004.tif]

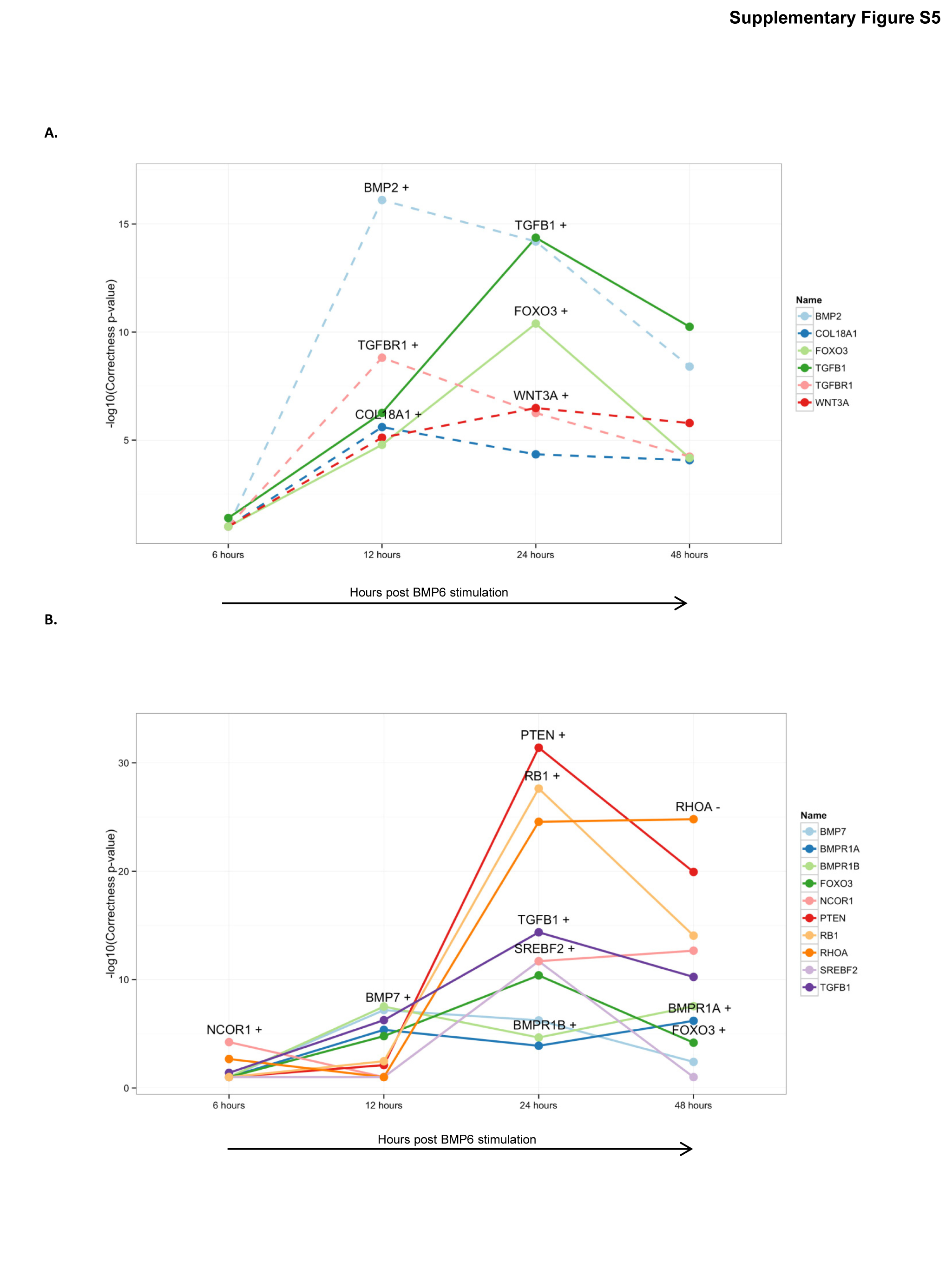

Supplement: Figure S5 — Causal Reasoning Engine (CRE) identifies potential causal drivers of BMP6 programmed C2C12 myoblast to brown preadipocyte-like switch. (TIF) [file pone.0092608.s005.tif]
