# Supplementary material for: Brown Fat Determination and Development from Muscle Precursor Cells by Novel Action of Bone Morphogenetic Protein 6
Source: PLoS One. 2014 Mar 21;9(3):e92608. doi: 10.1371/journal.pone.0092608 (PMC3962431; doi:10.1371/journal.pone.0092608)
Supplement: File S1 — Supporting information containing text for Figure S5, Table S5 and figure legends for all the supplementary figures. (DOC) [file pone.0092608.s006.doc]

**Supporting materials and methods:**

***Causal Reasoning Engine (CRE) Algorithm****-* The CRE algorithm employed here was introduced by Chindelevitch *et. al*. and provides statistical measures to assess relevance of upstream regulators to explain the observed expression changes. Briefly, the approach relies on a large collection of curated causal statements of the form:

*A [increases or decreases] B, where A and B are measurable biological entities.*

Here we focused only on changes in protein abundance or activity as upstream entities (A) and transcript levels as downstream entities (B). In total, we use approximately 300,000 causal statements of this type from commercial sources (Ingenuity Systems and Selventa). Each biological entity in the network and its assumed mode of regulation is a potential hypothesis. For each hypothesis, we can compare all possible downstream transcriptional changes in the knowledge base with the observed transcriptional changes in the experiment. The Correctness p-value is a measure of significance for the score of a hypothesis defined as (#correct – #incorrect). This score is high if the number of correct predictions exceeds the number of incorrect predictions. To ensure statistical significance under a null model of randomly re-assigning up- and down-regulated transcripts to arbitrary nodes, we computed the distributions for this score and derived appropriate p-values as described previously . CRE algorithm was applied to significantly regulated genes at the 6, 12, 24, and 48 hours post BMP6 stimulation and all upstream hypotheses that passed a Bonferroni corrected p-value of 0.05 for at least one of the time points were retained. The resulting 87 potential upstream drivers that are implicated in at least one time point are listed in Supplementary Table S5 (If accepted, production will need this reference to link the reader to the table).

**Supporting text for Supplementary Figure S5**

**Causal Reasoning Engine identifies additional potential drivers of BMP6 induced brown fat lineage commitment.**

To gain a better understanding of potential drivers of commitment response and to place Optn and Cox2 in context of known adipogenic differentiation pathway(s), Causal Reasoning Engine (CRE) analysis was employed. Briefly, CRE algorithm identifies potential upstream regulators of observed transcriptional changes based on a comprehensive knowledge base of previously identified regulatory relationships. If relevant biology is contained in the knowledge base of causal relationships, CRE is able to condense the thousands of differentially expressed genes across a time course experiment into fewer potential drivers of those downstream changes . Each resulting upstream hypothesis is associated with a p-value quantifying statistical significance of the prediction (Correctness p-value). This p-value takes into account the directionality of the previously observed relationships as well as the direction of regulation of the observed transcripts. Thus in order to elucidate the signaling upstream of Optn and Cox2, we investigated all hypotheses that correctly explain upregulation of Optn and Cox2 transcripts at the 12, 24, and 48 hours post BMP6 stimulation. This filter identified 2 hypotheses upstream of Optn (TGFβ1 and FOXO3) and 4 hypotheses upstream of Cox2 (BMP2, TGFβR1, COL18A1 and WNT3A) (Supplementary Figure S5A: If accepted, production will need this reference to link the reader to the figure). Interestingly, some of these hypotheses have previously been implicated in adipogenesis (BMP2 , COL18A1 , brown adipogenesis (TGFβ , WNT3A ). The two sets of hypotheses displayed distinct temporal patterns of activation with Cox2 hypotheses peaking at the 12 hour time point whereas the Optn hypotheses peaked at 24 hours. These data highlight the potential upstream mediators of Optn and Cox2 in the BMP6 induced commitment response and place Optn/Cox2 in context of signal transduction scheme known to be associated with adipogenesis. Next, in order to identify additional potential causal drivers of commitment phase, CRE analysis was applied to investigate potential upstream regulators that are active at different points during the BMP6 treatment time course. The hypotheses that passed Bonferroni-corrected p value of at least 0.05 for any time point were chosen. The full list of all the resulting potential regulators is presented in Table S5. As shown in Supplementary Figure S5B (If accepted, production will need this reference to link the reader to the figure), various causal hypotheses are altered at different time points that paint an overview of key regulators across the commitment time course. For example, BMP7 and BMPR1A gene signatures are induced at 12 hours. This is interesting as both BMP7 and BMPR1A have recently been implicated in brown fat differentiation . Along the same lines RB1, PTEN, SREBF2, RHOA, FOXO3, NCOR1, BMPR1B, TGFβ1 hypotheses are implicated at 24 hours. While RB1, PTEN, RHOA, NCOR1, TGFβ1 have previously been reported to regulate cell fate choice, lineage commitment, brown adipogenesis, mitochondrial biogenesis and energy expenditure in a context dependent manner , functional roles for BMPR1B and SREBF2 in context of brown fat differentiation remains obscure. Other interesting observation is the prevalence of NCOR1 activity at 24 hours and 48 hours post BMP6 stimulation. NCOR acts as a corepressor for PPARγ transcriptional activity and its displacement by Prdm16 in presence of SirT1 mediated deacetylation of PPARγ facilitates brown remodeling of white adipose tissue . Our data however reveal an unanticipated role for NCOR as being associated with BMP6 driven commitment of C2C12 cells. Interestingly, while all hypothesis displayed concordant directionality with BMP6 gene signature (as indicated by “+”), RHOA showed incongruous trajectory (indicated by “-”). In other words, RHOA repressed genes are significantly represented in BMP6 gene signature that peaks at 24 hours implicating inhibition of RHOA activity as an important causal driver of commitment phase. Taken together, these CRE generated analyses identify various potential causal drivers of BMP6 induced myoblast to brown preadipocyte-like commitment and place Optn and Cox2 in the context of adipogenic differentiation as studied in diverse model systems. Future studies will investigate the requirement of these hypotheses in facilitating BMP6 driven brown fat differentiation.

**References**

1. Chindelevitch L, Ziemek D, Enayetallah A, Randhawa R, Sidders B, et al. (2012) Causal reasoning on biological networks: interpreting transcriptional changes. Bioinformatics 28: 1114-1121.

2. Gutteridge A, Rukstalis JM, Ziemek D, Tie M, Ji L, et al. (2013) Novel pancreatic endocrine maturation pathways identified by genomic profiling and causal reasoning. PLoS One 8: e56024.

3. Pollard J, Jr., Butte AJ, Hoberman S, Joshi M, Levy J, et al. (2005) A computational model to define the molecular causes of type 2 diabetes mellitus. Diabetes Technol Ther 7: 323-336.

4. Enayetallah AE, Ziemek D, Leininger MT, Randhawa R, Yang J, et al. (2011) Modeling the mechanism of action of a DGAT1 inhibitor using a causal reasoning platform. PLoS One 6: e27009.

5. Macotela Y, Emanuelli B, Mori MA, Gesta S, Schulz TJ, et al. (2012) Intrinsic differences in adipocyte precursor cells from different white fat depots. Diabetes 61: 1691-1699.

6. Errera FI, Canani LH, Yeh E, Kague E, Armelin-Correa LM, et al. (2008) COL18A1 is highly expressed during human adipocyte differentiation and the SNP c.1136C > T in its "frizzled" motif is associated with obesity in diabetes type 2 patients. An Acad Bras Cienc 80: 167-177.

7. Yadav H, Quijano C, Kamaraju AK, Gavrilova O, Malek R, et al. (2011) Protection from obesity and diabetes by blockade of TGF-beta/Smad3 signaling. Cell Metab 14: 67-79.

8. Kang S, Bajnok L, Longo KA, Petersen RK, Hansen JB, et al. (2005) Effects of Wnt signaling on brown adipocyte differentiation and metabolism mediated by PGC-1alpha. Mol Cell Biol 25: 1272-1282.

9. Luo X, Hutley LJ, Webster JA, Kim YH, Liu DF, et al. (2012) Identification of BMP and activin membrane-bound inhibitor (BAMBI) as a potent negative regulator of adipogenesis and modulator of autocrine/paracrine adipogenic factors. Diabetes 61: 124-136.

10. Tseng YH, Kokkotou E, Schulz TJ, Huang TL, Winnay JN, et al. (2008) New role of bone morphogenetic protein 7 in brown adipogenesis and energy expenditure. Nature 454: 1000-1004.

11. Schulz TJ, Huang P, Huang TL, Xue R, McDougall LE, et al. (2013) Brown-fat paucity due to impaired BMP signalling induces compensatory browning of white fat. Nature 495: 379-383.

12. Li P, Fan W, Xu J, Lu M, Yamamoto H, et al. (2011) Adipocyte NCoR knockout decreases PPARgamma phosphorylation and enhances PPARgamma activity and insulin sensitivity. Cell 147: 815-826.

13. Qiang L, Wang L, Kon N, Zhao W, Lee S, et al. (2012) Brown remodeling of white adipose tissue by SirT1-dependent deacetylation of Ppargamma. Cell 150: 620-632.

14. Calo E, Quintero-Estades JA, Danielian PS, Nedelcu S, Berman SD, et al. (2010) Rb regulates fate choice and lineage commitment in vivo. Nature 466: 1110-1114.

15. Fernandez-Marcos PJ, Auwerx J (2010) pRb, a switch between bone and brown fat. Dev Cell 19: 360-362.

16. Scime A, Grenier G, Huh MS, Gillespie MA, Bevilacqua L, et al. (2005) Rb and p107 regulate preadipocyte differentiation into white versus brown fat through repression of PGC-1alpha. Cell Metab 2: 283-295.

17. Ortega-Molina A, Efeyan A, Lopez-Guadamillas E, Munoz-Martin M, Gomez-Lopez G, et al. (2012) Pten positively regulates brown adipose function, energy expenditure, and longevity. Cell Metab 15: 382-394.

18. Haas B, Mayer P, Jennissen K, Scholz D, Berriel Diaz M, et al. (2009) Protein kinase G controls brown fat cell differentiation and mitochondrial biogenesis. Sci Signal 2: ra78.

19. Mitschke MM, Hoffmann LS, Gnad T, Scholz D, Kruithoff K, et al. (2013) Increased cGMP promotes healthy expansion and browning of white adipose tissue. FASEB J 27: 1621-1630.

**Supplementary Figure Legends**

**Figure S1:** **Dose dependent response of BMP stimulation at driving adipogenic differentiation in C2C12 cells.**

**(A** and **B)** Q-PCR analyses of pan adipogenic markers (*Fabp4*, *Adiponectin*, *Perilipin4* and *HSL*) and *Ucp-1* in BMP6 or BMP7 (0.25, 2.5, 25 or 250ng/mL) pretreated cells followed by adipogenic differentiation and stimulation with 10μM forskolin (as outlined in Figure 1A). The expression in BMP untreated cells was set to 1, and results represent triplicate analyses of three independent biological replicates (mean + SD).

**Figure S2**: **BMP6 stimulated C2C12 cells display morphology indicative of non myogenic lineage commitment**.

C2C12 cells were stimulated with or without 250ng/mL of BMP6 or BMP7 for two days followed by sequential treatments illustrated in Figure 1A. Brightfield images (magnification: 10X) were captured after (i) 3 days in induction hormone cocktail media or (ii) after 4 days in post induction hormone cocktail media.

**Figure S3:** **Schematic of experimental strategy for transcriptional profiling study.**

**Figure S4: Achieved Cox2 and Optn knockdown does not diminish BMP6 induced *Elovl3* levels and differential effects of Cox2 selective inhibitor NS-398 on BMP6 induced brown fat differentiation in C2C12 cells.**

**(A)** Q-PCR generated Ct values for *Elovl3* transcript in BMP6 pretreated parental, shOptn and shCox2 cells followed by adipogenic differentiation. Each dot per condition represents an independent biological replicate. **(B)** Oil-Red-O staining in C2C12 cells stimulated with BMP6 and differentiated according to schematic shown in Figure 1A depicting time course of treatments. To investigate the effects of Cox2 inhibition, all treatments were supplemented with 0.1mM NS-398. Three representative images in absence or presence of NS-398 are shown. **(C)** Quantification of Oil-Red-O positive lipid droplets for panel B from eight random microfields. Error bars represent the standard error of the mean (**p<0.05*). **(D)** Q-PCR analyses of BAT markers (*Ucp-1, Cox7a1)* and pan-adipogenic marker (*Fabp4*) post two days of BMP6 pretreatment and adipogenic differentiation, in the presence or absence of NS-398 followed by 4 hours forskolin stimulation. Expression in NS-398 untreated cells was set to 1 and results represent triplicate analyses of two independent biological replicates (mean + SD), **p<0.05.* Similar results were achieved in 2 independent analyses*.*

**Figure S5: Causal Reasoning Engine (CRE) identifies potential causal drivers of BMP6 programmed C2C12 myoblast to brown preadipocyte-like switch.**

**(A)** All CRE generated hypotheses that correctly explain changes in *Optn* and *Cox2* transcript levels at 12, 24 and 48 hours post BMP6 stimulation are depicted. Hypotheses explaining *Optn* are represented by solid lines while hypotheses that explain observed changes in *Cox2* are shown by dotted lines. **(B)** Selected CRE hypotheses that are significant for at least one time point in the BMP6 stimulated time course cascade. Y-axis is based on the “Correctness p-value” with higher values implying more significant findings at that time point.
